# A minimal physiologically based pharmacokinetic model to study the combined effect of antibody size, charge, and binding affinity to FcRn/antigen on antibody pharmacokinetics

**DOI:** 10.1007/s10928-023-09899-z

**Published:** 2024-02-24

**Authors:** Krutika Patidar, Nikhil Pillai, Saroj Dhakal, Lindsay B. Avery, Panteleimon D. Mavroudis

**Affiliations:** 1 Department of Chemical and Biological Engineering, University at Buffalo, The State University of New York, Buffalo, NY USA; 2 Global DMPK Modeling & Simulation, Sanofi, 350 Water St, Cambridge, MA 02141 USA; 3 Global DMPK Innovation, Sanofi, Cambridge, MA USA

**Keywords:** Monoclonal antibody, Minimal PBPK, PBPK, Size-based transport, Tissue disposition, Two-pore model

## Abstract

Protein therapeutics have revolutionized the treatment of a wide range of diseases. While they have distinct physicochemical characteristics that influence their absorption, distribution, metabolism, and excretion (ADME) properties, the relationship between the physicochemical properties and PK is still largely unknown. In this work we present a minimal physiologically-based pharmacokinetic (mPBPK) model that incorporates a multivariate quantitative relation between a therapeutic’s physicochemical parameters and its corresponding ADME properties. The model’s compound-specific input includes molecular weight, molecular size (Stoke’s radius), molecular charge, binding affinity to FcRn, and specific antigen affinity. Through derived and fitted empirical relationships, the model demonstrates the effect of these compound-specific properties on antibody disposition in both plasma and peripheral tissues using observed PK data in mice and humans. The mPBPK model applies the two-pore hypothesis to predict size-based clearance and exposure of full-length antibodies (150 kDa) and antibody fragments (50–100 kDa) within a onefold error. We quantitatively relate antibody charge and PK parameters like uptake rate, non-specific binding affinity, and volume of distribution to capture the relatively faster clearance of positively charged mAb as compared to negatively charged mAb. The model predicts the terminal plasma clearance of slightly positively and negatively charged antibody in humans within a onefold error. The mPBPK model presented in this work can be used to predict the target-mediated disposition of a drug when compound-specific and target-specific properties are known. To our knowledge, a combined effect of antibody weight, size, charge, FcRn, and antigen has not been incorporated and studied in a single mPBPK model previously. By conclusively incorporating and relating a multitude of protein’s physicochemical properties to observed PK, our mPBPK model aims to contribute as a platform approach in the early stages of drug development where many of these properties can be optimized to improve a molecule’s PK and ultimately its efficacy.

**Supplementary Information:**

The online version contains supplementary material available at 10.1007/s10928-023-09899-z.

## Introduction

Antibodies represent a diverse array of compounds that have revolutionized the treatment of a wide range of diseases. About 350 new antibody entities are in active clinical development [1]. Antibodies have several advantages over small-molecule drugs such as high specificity, high tolerability, and longer half-lives. Despite recent progress, only a small percentage of compounds in development enter the clinic and ultimately reach the market, often due to lack of desired pharmacological activity [2]. Current research in drug design and development is focused on identifying factors responsible for improved pharmacological activity of antibodies. During drug development, drug candidates are often iteratively studied for key pharmacokinetic (PK) parameters associated with absorption, distribution, metabolism, and excretion (ADME) [3, 4]. Monoclonal antibody (mAb) pharmacokinetics is being extensively studied with additional focus on drug’s distribution and elimination properties. Among these, neonatal Fc receptor (FcRn) binding and molecular size have been shown to significantly affect the systemic PK of antibodies [5]. However, characteristics such as antibody charge and affinity to interact non-specifically with charged cell components are among a few properties that are yet to be studied extensively. A relationship between the physicochemical properties of antibodies such as molecular weight, size, charge, FcRn binding affinity, and its PK response is still largely unknown but remains crucial for establishing an initial understanding of a drug’s PK and desired target engagement.

Physiochemical properties including molecular weight, size (Stokes radius), charge, hydrophobicity, binding affinities, isoelectric point (pI), and glycosylation can highly influence mAb PK [1, 6, 7]. The molecular weight and size of the antibody affects its absorption properties and is an important physicochemical characteristic for subcutaneously administered antibodies [6]. An accurate quantitative relationship between molecular weight or size of an antibody and the fraction absorbed has been challenging to find, particularly across species [6]. Typically, systemically administered antibodies exhibit relatively fast distribution followed by a slower elimination phase, which is attributed to their relatively large size, surface charge, long half-lives, and affinity to bind to FcRn receptor [1, 6, 8]. The distribution in tissues is facilitated by the movement across the vascular barrier into the interstitial fluid spaces either by crossing the endothelial cell barrier or the paracellular pores. This movement across the endothelial cell membranes is often limited by the size and charge of antibodies [6]. The molecular size of antibodies affects their distribution, which restricts them primarily in vascular and interstitial spaces in tissues, leading to a relatively smaller volume of distribution [7]. Several PBPK models have incorporated size-based transcapillary movement of antibodies using a two-pore hypothesis and derived transport parameters [9–12]. Physicochemical properties can have an impact on clearance and half-life of antibodies. Unlike small molecule drugs, the elimination of antibodies through the kidney is considered insignificant, since typically monoclonal antibodies (mAbs) have a much higher molecular mass and size and are restricted by the glomerular filtration barrier [6, 13]. For large molecule drugs, such as mAbs or antibody fragments (Fabs) that are larger than albumin (66–67 kDa), they undergo no or very limited renal clearance [14, 15]. Generally, mAbs are eliminated through lysosomal mediated proteolytic degradation that results in smaller peptides and amino acids [1, 3]. Antibodies are cleared from circulation via different specific or non-specific clearance mechanisms such as, (i) non-specific clearance via cell pinocytosis, (ii) proteolytic degradation within lysosomes, (iii) catabolism, and (iv) antigen-mediated specific clearance [1, 3, 4].

Another vital characteristic of an antibody is its affinity to FcRn receptor and specific antigens. Briefly, mAb binds to the FcRn receptor at a slightly acidic pH, which protects antibodies from proteolytic degradation in the lysosomes and allows recycling of the antibody back into circulation [7, 8]. Garg and Balthasar showed that FcRn binding Immunoglobulin G (IgG) in wild-type mice had relatively lower plasma clearance compared to IgG administered in FcRn knockout mice [16]. Chang et al. reported clear differences in distribution and clearance of a wild-type IgG compared to a FcRn non-binding IgG in mice [17]. Rafidi et al. assessed the effect of both antibody size and FcRn binding affinity on systemic pharmacokinetics and tissue distribution [5]. The FcRn non-binding IgG fragments did not reach steady state kinetics in most tissues when compared to full length FcRn binding IgG [5]. Moreover, de Witte et al. also validated a strong sensitivity to FcRn receptors through a PK-Sim model for large molecules in plasma pH, and provided necessary mechanistic adaptations to account for such molecules especially in the context of FcRn internalization and recycling [8]. On the other hand, mAbs also bind to a specific antigen, and as a result their ADME characteristics may change based on the binding affinity of antibody-antigen interaction, antigen density, rate of internalization into the cells, and rate of degradation of the complex [1].

The net surface charge, charge distribution, and pI of an antibody affect nonspecific cellular uptake and degradation [18]. The surface charge of a therapeutic protein is a property of the amino acid sequence of the protein and the pH of its surroundings [6]. Most therapeutic proteins have an isoelectric point (pI) in the range of 5–9. Most antibodies are slightly positively charged with pI values between 7 and 9.10 [6]. The net surface charge is hypothesized to lead to non-specific interactions with the charged extracellular matrix components and membrane proteins in the cells, resulting in enhanced pinocytotic uptake and degradation, suggesting that charge can be a relevant descriptor to potentially predict antibody ADME properties [7]. Igawa et al. showed that lowering the total pI of an antibody, a property often related to antibody charge, resulted in longer half-life and slower elimination rate [19]. Bumbaca Yadav et al. also demonstrated that modifying surface charge and hydrophobicity altered mAb PK [20] A more positively charged antibody variant showed a relatively faster non-specific clearance than a less positively charged variant of the antibody [20]. In contrast, some studies reported no correlations between clearance and pI/charge of an antibody, especially when extremely negative or positive charged variants were analyzed [18, 21]. Several underlying mechanisms explaining this phenomenon have been hypothesized such as altered FcRn binding, differential stability, increased hydrophobicity, and different catabolic activity of some tissues [18]. There are ongoing efforts to understand the relationship between charge and antibody PK through experimentation and computational modeling. Recently published PBPK models correlate antibody charge and ADME properties based on essential processes like pinocytosis, nonspecific clearance, volume of distribution [22, 23]. At present, the optimization of these determining factors for better antibody distribution is being explored. Additionally, the influence of other compound-specific properties such as their binding affinity to specific antigens, hydrophobicity, viscosity, chemical stability, and tissue-specific features such as membrane structure and blood flow on ADME in plasma and different tissues is being explored as well [1, 21].

In this work, we incorporate the combined quantitative effect of molecular weight, size, charge, and non-specific binding to both FcRn and tissue constituents, and specific antigen binding of large molecule drugs on their PK using a minimal physiologically-based pharmacokinetic (mPBPK) model. In model development, we derive empirical equations to quantitatively relate the compound-specific properties to the PK parameters. Our model incorporates specific clearance of large molecule drugs through target-mediated drug disposition in both plasma and tissues, and non-specific clearance through cell pinocytosis, non-specific charge-based interactions, lysosomal degradation, and cellular catabolism. We fitted and validated our model using published PK data for the following cases, non-specific FcRn-binding and FcRn non-binding mAbs, specific FcRn binding mAbs, different sized mAbs and antibody fragments, and different charge-variants of mAbs. By incorporating these essential processes, we aim to use our mPBPK model and establish a quantitative link between PK and different large molecule physicochemical characteristics, a relationship that can be crucial in early optimization of large molecules.

## Methods

### Model structure

**Fig. 1:**
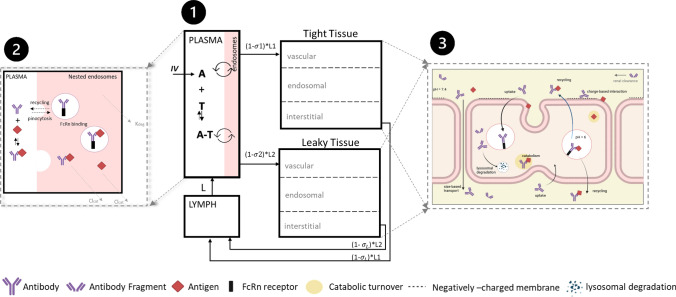
A schematic diagram of the minimal PBPK model developed. Minimal PBPK model (**1**) consists of plasma, lymph, tight tissue, and leaky tissue compartments. Each tissue compartment is divided into vascular, endosomal, and interstitial space as shown in the right (**3**). The plasma compartment is nested with endosomal space present in the systemic vascular endothelial cells. Monoclonal antibody (A) can intravenously (IV) administered in the plasma compartment. mAb (A) interacts with antigen (T) to form an antibody-antigen complex (A-T). mAb (A), soluble antigen (T), and complex (A-T) enter the nested endosomes in plasma through periodic pinocytosis/exocytosis processes (**2**). Free mAb (A) and mAb-antigen complex (A-T) binds to soluble and membrane antigens (red diamonds), undergoes pinocytosis into the endothelial cells, binds to FcRn (black rectangle) interacts with negatively charged membrane proteins (dashed line), and transports through the paracellular pores based on their size. In the endosomal space, mAb binds to FcRn and soluble target receptors at slightly acidic pH (pH = 6). Free mAb in the endosomes degrades through lysosomal degradation. Unbound antigen and mAb-antigen complex catabolize within endosomes. FcRn-bound mAb and FcRn-bound mAb-antigen complex are recycled back to vascular and interstitial space of the tissues. mAb undergoes size-based renal clearance in leaky tissue compartment. are vascular reflection coefficients. L1 and L2 are lymph flow rates in the tight and leaky tissue, respectively. L is the lymph flow rate. A representation of nested endosomes in the plasma compartment is provided (**2**) Antibody (Y-shape) interacts with soluble targets (red diamond) in plasma compartment to form antibody-receptor complex. In the nested endosomes, antibody freely interacts with soluble targets and FcRn receptor (black rectangle) at pH = 6. The unbound antibody degrades at a rate *.* The soluble receptors and antibody-receptor complex are cleared through catabolism at a clearance rate, *.* Subfigure (**3**) created with BioRender.com (Color figure online)

The structure of the mPBPK model, a schematic diagram of which is shown in Fig. 1, is mainly based on previously developed minimal and full PBPK models [11, 24–27]. It comprises plasma, lymph, and two lumped tissue compartments, tight and leaky. Tight tissue represents muscle, fat, brain, and skin, whereas leaky tissue the rest of the body tissues. The tissues with similar kinetics are lumped into respective tight or leaky compartments as previously proposed by Cao et al. [26]. As reported by Sarin et al., the blood capillary types in different tissues and organs can be distinguished based on the upper limit of pore size to the transvascular flow of large molecules [28]. The tissues with upper limit of capillary pore size approximately below 5 nM (brain, muscle, skin, and adipose) were lumped together as a compartment, and all other tissues were lumped as another compartment [28]. Arterial blood flow to tight/leaky tissue compartments is dependent on the vascular reflection coefficient for each tissue (${\sigma }_{1}, {\sigma }_{2}$). The lymph flow out of each tissue compartment is collected in the lymph compartment, which is dependent on the lymphatic reflection coefficient (${\sigma }_{L}$). The lymph flow rate to each tissue compartment (${L}_{1}, {L}_{2}$ ) is calculated as a sum of individual tissue lymph flow rates reported previously [27]. Lymph flow from tight/leaky tissues is delivered back to plasma via the lymph compartment. Each tissue is divided into vascular, endosomal, and interstitial spaces. The unbound drug and drug-antigen complex drains from the interstitial space into the lymph compartment and is delivered back to the plasma and the systemic circulation. In our model, plasma is nested with an endosomal sub-compartment (Fig. 1), which represents endothelial cells in the systemic vascular space as discussed in previous models [24, 29]. Endothelial cells found in blood vessels can be different from the ones present in the organs, which add another dimension of complexity to our model [29].

### Model dynamics

#### FcRn/antigen binding

The free mAb binds to the soluble antigen in plasma to form a soluble drug-antigen complex. The free mAb, antigen, and the mAb-antigen complex are taken up via pinocytosis into the nested endosomal sub-space in plasma, where free mAb and mAb-antigen complex can bind to FcRn receptor at slightly acidic pH (pH = 6) in the endosomes. The model assumes that available FcRn concentration in tissue and plasma endosomes remains same but varies with species [27] whereas the administered mAb are assumed to bind to FcRn at only slightly acidic, pH = 6, in the endosomes [24]. The FcRn-bound mAb and FcRn-bound mAb-antigen complex are recycled back to plasma and salvaged from lysosomal degradation. The unbound mAb within the endosomes is degraded via a first-order rate constant by lysosomal degradation, whereas unbound antigen and mAb-antigen complex is catabolized in the endosomes. The unbound mAb and mAb-antigen complex circulate to the tissue compartments and enter the vascular sub-space in the tissues. The unbound drug can bind competitively with soluble and membrane-bound antigens. The soluble targets are assumed to be synthesized both in plasma and in vascular space in the tissues, whereas membrane-bound targets are assumed to be synthesized in the tissue vascular space [24]. The membrane-bound mAb-antigen complex is assumed to internalize at the same rate as the rate of antigen degradation as drug often times does not affect complex internalization [1]. The drug target complex and unbound target are assumed to be catabolized at the same rate ($C{L}_{cat}$) [24]. The mAb-antigen complex internalized in the tissues, and the internalized molecules are assumed to be metabolized further but were not explicitly modeled. The model does not account for a cellular space to describe downstream processes, and remains a current limitation of the model. The soluble mAb-antigen complex is taken up by tissue endosomes via pinocytosis. In the tissue endosomes, the unbound mAb and mAb-antigen complex interact with FcRn. The FcRn-bound mAb and FcRn-bound mAb-antigen complex are recycled back to both vascular space and interstitial space of the tissues.

#### Size-based transport

In our model, the unbound mAb present in the tissue vascular space transports to the interstitial sub-space in the tissues based on the two-pore model. Rippe and Haraldsson proposed the two-pore theory to study the transcapillary movement of large molecules through the paracellular pores in the tissues [30]. The tissue vasculature is assumed porous, and the radius of pores is loosely classified into small pores (40 nm) and large pores (220 nm) [10]. Large molecule drugs are transported through these two sets of pores in a size-dependent manner. The two-pore theory has been successful in explaining the extravasation of antibodies into the interstitial space of the tissues previously [9, 10, 30, 31]. Li and Shah presented the two-pore PBPK model, using de novo derived parameters, to predict the plasma PK of different sized proteins (13–150 kDa) without any parameter estimation [10]. Our mPBPK model includes size-based transport of large molecule drugs through small and large pores. The parameters such as permeability-surface area product, Peclet number, vascular reflection coefficients, fractional tissue lymph flows through pores are dependent on molecular weight and molecular size of the antibody and are calculated using derived equations (eqs. A40–A48) in [10].

#### Charge-based interaction

The charge-dependent effect on PK is reflected in the mPBPK model in the following processes. First, the charge variations of mAb affect the rate of pinocytosis or uptake into the endosomes, which is accommodated by multiplying a scaling factor (${S}_{pino}$) with the pinocytosis rate [22, 32]. Second, the unbound mAb undergo non-specific interactions with negatively charged cell membrane proteins (${{\text{R}}}_{{\text{m}},{\text{total}}}$) in the tissue vasculature [22, 33]. The total concentration of membrane protein receptor (${R}_{m,total}$) is assumed fixed across tissues [22]. The unbound mAb with a net positive charge is assumed to have high affinity for negatively charged cellular components, whereas mAb with a net negative charge is assumed to have lower affinity for cellular components due to repulsive forces between them. The non-specific binding between mAb and membrane proteins is characterized by an association rate constant (${k}_{on,NSB}$), which is assumed equal to the mAb-FcRn association rate constant (${k}_{1on}$) [33], and a dissociation rate constant (${k}_{off,NSB}$). Third, slight positive or negative charge on the mAb affect the volume of distribution in the interstitial space in the tissues [25, 34]. The change in volume of distribution for charge-variants is included in the model by multiplying a scaling factor (${K}_{p}$) to the volume of interstitial space [23, 25].

### Model Parameters

Our mPBPK model structure remains the same across species. The model equations are provided in the Supplementary File, eq. A1–A39. The model parameters for the mPBPK model are loosely divided into two groups, physiology-based parameters and physicochemical or compound-based parameters. Additionally, some compound-specific parameters like molecular weight, size, and charge are related to model parameters through derived and empirical equations. The compiled list of physiology-based parameters is given in Table 1 in the Supplementary File. The physiological parameters such as body weight, volumes, flow rates etc. is dependent on the species of interest and adopted from respective literature sources [24–27]. The kinetic rate constant parameters such as pinocytosis rate, FcRn recycling rate, catabolic rate are assumed same across tissue compartments [27], unless stated otherwise. The kinetic rate constants such as endosomal uptake rate (${k}_{up}, {k}_{up,p}$) were estimated among a range provided previously [27]. The endosomal recycling rate ($C{L}_{rec}$) for nested endosomes in plasma and tissue endosomes were calculated using endosomal transit time (8 min) and volume of endosomes in mice as discussed by Yuan et al. [24]. The target-specific parameters such as baseline expression ($IC{C}_{pT}$), half-life ($C{L}_{pT}$), and internalization rate (${k}_{int}$) are considered in the model. The rate of synthesis of soluble and membrane-bound antigens is calculated as a product of baseline concentration and half-life of the antigen. The baseline concentration of soluble and membrane-bound antigens was obtained from previous studies [35, 36]. The half-life of the soluble antigens is assumed to be 2 h based on [36], and half-life of membrane-bound antigens is estimated between 10 and 40 h [36, 37].

The compound parameters vary with drug’s physicochemical properties. In our mPBPK model, molecular weight (${\text{MW}}$ ) of drug, Stoke’s radius (${a}_{e}$) of drug, net surface charge on drug, fraction of interstitial volume available (${K}_{p}$) for drug, association rate and dissociation rate constants for antibody-FcRn binding at slightly acidic pH (pH = 6) (${k}_{1on}, {k}_{1off}$), association rate and dissociation rate constants for antibody-antigen binding at physiological pH (pH = 7.4) (${k}_{on}, {k}_{off}$) and slightly acidic pH (pH = 6) (${k}_{eon}, {k}_{eoff}$), and dissociation rate constant for non-specific drug interactions are considered (${K}_{D, NSB}$). ${k}_{1on}\mathrm{ and }{k}_{1off}$ values for antibody-FcRn binding in acidic pH and ${k}_{on} , {k}_{off} ,{k}_{eon},\mathrm{ and }{k}_{eoff}$ values for antibody-antigen binding are obtained from published experiments [22, 35]. These compound-specific properties of the drug are provided in Table 1 in the Supplementary File.

The derived and empirical equations are used to incorporate the effect of MW, size, and charge on antibody pharmacokinetics. Li and Shah have previously incorporated size-dependence based on a two-pore hypothesis [10]. The two-pore theory provides transport equations to derive parameters such as re-circulation rate, fluid flow rate through different sized pores, vascular reflection coefficients for different sized pores, permeability-surface area product, Peclet number etc., which are used in the model to describe the size-based transcapillary movement of antibodies through paracellular pores without any parameter estimation. Additionally, a renal clearance parameter ($C{L}_{renal}$) is empirically-derived using a quantitative relation between glomerular sieving coefficient and Stoke’s radius of an antibody (Fig. A2). The fixed values of size-dependent model parameters are given in Table 2. In our model, effect of charge on mAb PK is included through a set of empirically-derived parameters (${K}_{D,NSB}, {K}_{p}, {S}_{pino}$) and relate them to the antibody charge (Fig. A3-A4). We provide additional detail on equations and methodology used to derive these parameters in the Model Development and Supplementary File.

### Model development

The proposed mPBPK model was fitted and validated using published experimental data from multiple sources. We performed model fitting only where necessary as most model parameter values are known a priori. The mPBPK model parameters were fitted against published data for the following cases, (1) non-specific IgG antibody binding to FcRn in mice (${k}_{up}, {k}_{up,p}, {\sigma }_{1},\mathrm{ and} {\sigma }_{2}$) [16], (2) non-specific IgG antibody binding to FcRn knockout mice (${k}_{up}$) [16], (3) charge-variants of non-specific IgG with an intact Fc region (${{R}_{m,total},K}_{D,NSB}, {S}_{pino}, {K}_{p}$) [22], and (4) anti- carcinoembryonic antigen (CEA) IgG in mice (${k}_{p,Tm}, {k}_{int}$) [37]. The mPBPK model was evaluated using the fitted and derived parameters for the following cases, (1) non-specific IgG antibody binding to FcRn in mice [38], (2) size-variants of non-specific IgG with or without intact Fc region, (3) charge-variants of non-specific IgG with an intact Fc region [18, 39]. To compare the predicted concentration in plasma and tissues, we digitized observed data for each case from published sources using *WebPlotDigitizer* [40]. Most studies report observed concentrations in individual tissues. The concentration in different sub-spaces in the tissue such as vascular, interstitial, or endosomal space were often not measured. Therefore, we calculated observed total tight tissue concentration by adding the observed drug amount in brain, muscle, skin, and fat tissues and dividing the sum by total tight tissue volume. The observed total leaky tissue concentration was calculated in a similar manner for other tissues. For each case, we calculated the total predicted tight tissue and leaky tissue concentrations using Eqs. 1 and 2, where ${C}_{e1}, {C}_{e2}$ are endosomal concentrations, ${C}_{v1}, {C}_{v2}$ are vascular concentrations, ${{\text{C}}}_{{\text{is}}1}, {{\text{C}}}_{{\text{is}}2}$ are interstitial concentrations in tight and leaky tissue compartments, respectively. We acknowledge that the total tissue concentration of antibodies may not be the most appropriate to correlate with therapeutic effect of an antibody [41]. However, model development for each scenario was limited by the available published data. The total tissue concentrations were compared against observed total tissue concentrations in leaky and tight tissues. The prediction error between predicted and observed plasma and tissue concentration is calculated as a sum of squared error (SSE) normalized by the mean of the observed data. For parameter estimation, we used a multi-start non-linear least squares optimization method (*fmincon*) in MATLAB, where 10 starting values for each parameter were sampled using Latin hypercube sampling method in MATLAB. The best fitted parameters are obtained based on the least SSE between prediction and data.

1 $${\text{C}}_{{{\text{tight}},{\text{ total}}}} \, = \,\frac{{({\text{C}}_{{{\text{e}}1}} {\text{*V}}_{{{\text{e}}1}} + {\text{C}}_{{{\text{v}}1}} {\text{*V}}_{{{\text{v}}1}} + {\text{C}}_{{{\text{is}}1}} {\text{*V}}_{{{\text{is}}1}} )}}{{{\text{V}}_{{{\text{e}}1}} + {\text{V}}_{{{\text{v}}1}} + {\text{V}}_{{{\text{is}}1}} }}$$

2 $${\text{C}}_{{{\text{leaky}},{\text{ total}}}} \, = \,\frac{{({\text{C}}_{{{\text{e}}2}} {\text{*V}}_{{{\text{e}}2}} + {\text{C}}_{{{\text{v}}2}} {\text{*V}}_{{{\text{v}}2}} + {\text{C}}_{{{\text{is}}2}} {\text{*V}}_{{{\text{is}}2}} )}}{{{\text{V}}_{{{\text{e}}2}} + {\text{V}}_{{{\text{v}}2}} + {\text{V}}_{{{\text{is}}2}} }}$$

#### Local sensitivity analysis

First, we performed a local sensitivity analysis on the mPBPK model to obtain the most sensitive parameters. Each parameter value was increased by 20% from its original value, and a relative percent change ($\mathrm{\Delta AUC }\left(\mathrm{\%}\right)$) in plasma and tissue exposure (AUC) was calculated as shown in Eq. 3, where ${{\text{AUC}}}_{{\text{p}},+20\mathrm{\%}}$ is AUC calculated with perturbed parameter value, and ${{\text{AUC}}}_{{\text{p}}}$ is AUC calculated with original parameter value. Fig. A1 in the Supplementary File shows the relatively sensitive parameters and respective change in AUC. The perturbed parameter that causes a relatively higher change in exposure is considered more sensitive. The most sensitive parameters were ${k}_{up}, {k}_{up,p}, {\sigma }_{1},\mathrm{ and} {\sigma }_{2}$ in the model and were fitted to the non-specific FcRn binding IgG1 dataset [16].

3 $$\Delta {\text{AUC }}\left( {\text{\% }} \right)\, = \,\frac{{{\text{AUC}}_{{{\text{p}}, + 20{\text{\% }}}} - {\text{AUC}}_{{\text{p}}} }}{{{\text{AUC}}_{{\text{p}}} }} \times 100$$

#### Model fitting and validation

For Case (1), we fitted the model to a non-specific IgG1 dataset in mice, where IgG1 does not bind to a specific target, here target-mediated specific clearance was not included in the model. Case (1) was validated using a validation dataset for non-specific IgG1 in mice [38]. Case (2) demonstrates the effect of FcRn knockout on IgG PK in plasma and tissues. Here, we recalibrated the rate-limiting parameter ${k}_{up}$ to capture the faster clearance of IgG in absence of FcRn receptor in mice [16].

The effect of size of an antibody was reflected in the model using a transcapillary two-pore clearance from vascular to interstitial space in tissues, and a size-based renal clearance term. The size-based clearance through large pores and small pores is calculated using derived equations based on the two-pore hypothesis (eqs. A47–A48) [10]. For smaller sized antibody fragments with antibody size less than 4 nm, renal clearance plays an important role in non-specific clearance of an antibody [11]. The size-based renal clearance (L/h) is calculated using Eq. 4, where *GFR* is the glomerular filtration rate of kidneys in mice (L/h), and *ϴ* is the sieving coefficient. We fitted an empirical relationship between *ϴ* and Stoke’s radius (${a}_{e}$) (Fig. A2) using data provided by Haraldsson et al. [42]. For larger antibodies (> 4 nm), clearance through kidney is insignificant [10]. The effect of size of various IgG and IgG fragments on plasma and tissue PK was validated using published data for non-specific IgG ranging from 50 to 150 kDa. For this case, no model fitting was necessary.

4 $${\text{CL}}_{{{\text{renal}}}} \, = \,{\text{GFR}} * {\uptheta }$$

The effect of charge on antibody PK is reflected in the model through a modulation factor (${K}_{p}$) for interstitial volume of distribution, a modulation factor (${S}_{pino}$) for pinocytosis rate, and a charge-dependent non-specific equilibrium dissociation constant (${K}_{D, NSB}$). Previous PBPK models have related charge with pinocytosis [22, 32, 43] and non-specific interactions [4, 22, 33]. Recent work by Liu et al. provided a good description of surface charge modifications on antibody disposition [18]. In their work, the complementarity determining regions (CDRs) of an antibody were systematically engineered to create a series of variants with an isoelectric point (pI) range of 6.3–8.9 that had a variable Fv charge identified using protein sequences [18]. Liu and Shah used part of this dataset to model the effect of charge on pinocytosis and non-specific interactions [22]. In the mPBPK model, we use this dataset to incorporate the effect of charge on volume of distribution in addition to pinocytosis and non-specific interactions. We use this published data for three different charge-variants of an antibody (150 kDa) to fit ${S}_{pino}$, ${K}_{p},$ and ${K}_{D, NSB}$ parameters in the mPBPK model. The neutral IgG variant had a zero net surface charge, a positive IgG variant had a + 5 net surface charge, and negative IgG variant had a -8 net surface charge. The non-specific interactions between an IgG and negatively-charged membrane proteins (${R}_{m,total}$) are incorporated using a dynamic set of equations, where concentration of total membrane protein receptor (${R}_{m,total}$) is assumed fixed. ${R}_{m,total}$ is estimated in our model for the neutral IgG dataset [22], and kept constant for other charge-variants of IgG. ${K}_{p}$ is set to 1 for neutral IgG and estimated for slightly positive and slight negative charge-variants of IgG. We fixed ${S}_{pino}$ to 1 in nested endosomes in plasma, as charge did not affect IgG uptake in nested plasma endosomes. The modulation factor ${S}_{pino,1}$ for pinocytosis rate into the tight tissue endosomes and ${S}_{pino,2}$ for pinocytosis rate into the leaky tissue endosomes are set to 1 for neutral IgG. ${S}_{pino,1}$ was fixed to 1 for positively-charged IgG, based on previously reported values for tight tissues [22]. We estimated ${S}_{pino,2}$ in leaky tissue endosomes for positively charged IgG. The value of ${S}_{pino,1}\mathrm{ and }{S}_{pino,2}$ is assumed to be 1 for negatively-charged IgG based on previously reported values [22]. We estimated the equilibrium dissociation constant (${K}_{D, NSB}$) for positive, neutral, and negative charge-variants of IgG using tissue and plasma PK data [22]. Using the estimated and known parameter values of ${K}_{p}$ and ${K}_{D, NSB}$ for respective charge-variants of mAb, we derived an empirical relationship between ${K}_{D, NSB}$ and surface charge (Fig. A3), and ${K}_{p}$ and surface charge (Fig. A4) using curve fitting in MATLAB. The choice of a numerical function to fit these parameters was based on goodness-of-fit and SSE. These charge-dependent effect on PK parameters were validated using a separate but limited dataset for − 4, − 10, and + 10 charge variants of an antibody [18, 39].

To account for target-mediated effects on PK, we simulated the model response in presence of membrane-bound antigens using available data for anti-CEA IgG administered intravenously at three different doses in mice (1 mg/kg, 10 mg/kg and 25 mg/kg [37]). The model was simulated to a priori model parameters such as baseline membrane-bound antigen concentration (${T}_{m0}$) and antigen half-life for membrane-bound receptors. Antibody-antigen association (${k}_{on}$) and dissociation rate (${k}_{off}$) constant were assumed same for soluble and membrane-bound antigens [35]. The internalization rate (${k}_{int}$) of membrane-bound antigen was initially assumed equal to the degradation rate of membrane-bound antigen. However, we found that synthesis rate of membrane-bound antigens (${k}_{syn,m}$), degradation rate of membrane-bound antigens (${k}_{p,Tm}$),${k}_{on}$, ${k}_{off}$, and ${k}_{int}$ were sensitive parameters. The synthesis rate of membrane-bound antigens is the product of the degradation rate and baseline concentration of the membrane-bound antigens. The baseline concentration of membrane-bound CEA antigen and ${k}_{on}$ and ${k}_{off}$ values for antibody-antigen binding are obtained from previous experimental studies [35, 37]. The degradation rate of membrane-bound antigen is obtained from the half-life of the membrane-bound antigens. In previous studies, it was assumed that the half-life of the membrane-bound CEA antigen was between 10 and 17 h and the internalization rate of CEA antigen was equal to the degradation rate of the antigen [37]. Therefore, we only optimized the internalization rate of membrane-bound mAb-antigen complex and degradation rate of membrane-bound antigens (${k}_{p,Tm}$) and kept the other sensitive parameters at their original reported values. We used the dataset for anti-CEA IgG administered at a low dose of 1 mg/kg to estimate these parameters [37]. We validated the target-mediated model response for two separate datasets, where anti-CEA IgG administered at 10 mg/kg dose and 25 mg/kg dose [37].

## Results

### Non-specific antibody PK in mice

**Fig. 2:**
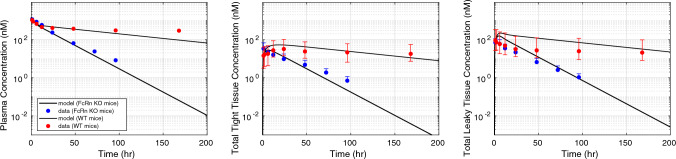
Pharmacokinetic (PK) response of non-specific IgG predicted in wild-type mice (red) and FcRn knockout mice (blue). The model fitted (solid black curve) concentration in plasma (left), tight (middle), and leaky (right) tissue compartments after intravenous administration of 8 mg/kg dose is shown. The observed concentration of IgG in wild-type mice is shown as solid red points [16]. The observed concentration of IgG in FcRn knockout mice is shown as solid blue points [16]. The data intervals are min–max interval obtained from the individual tissue concentrations (Color figure online)

**Fig. 3:**
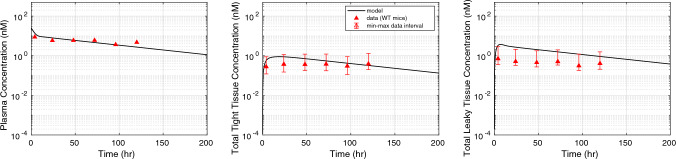
Pharmacokinetic (PK) response of non-specific IgG validated in wild-type mice (red). The model predicted (solid black curve) concentration in plasma (left), tight (middle), and leaky (right) tissue compartments after intravenous administration of 3.8 µg/kg dose is shown. The observed concentration of IgG in wild-type mice is shown as solid red points [38]. The data intervals are min–max interval obtained from the individual tissue concentrations (Color figure online)

A local sensitivity analysis identified the most sensitive model parameters and reduced the parameter space for parameter estimation. The effect of change in parameter values on the relative percent change in plasma, tight tissue, and leaky tissue exposure (AUC) is shown in Fig. A1 in the Supplementary File. The pinocytosis rate into the tissue endosomes (${k}_{up}$) and plasma endosomes (${k}_{up,p}$), and vascular reflection coefficients (${\sigma }_{1} {\text{and}} {\sigma }_{2}$) are most sensitive, and their values were optimized against the non-specific IgG1 dataset [16]. The tissue lymph flow into leaky tissues (${L}_{2}$) is a sensitive physiological parameter, however its value is assumed fixed, and is a sum of reported values for individual tissue lymph flow rates for mice (28 g) [27]. The fitted model response (Fig. 2) for a non-specific IgG dataset (IV dose 8 mg/kg) captures the plasma and lumped tissue concentrations for a non-specific FcRn binding IgG in mice. The estimated uptake rate for nested endosomes in plasma (${k}_{up,p}$) and tissue endosomes (${k}_{up}$) is 0.05 1/h and 0.0276 1/h, respectively, which is close to previously predicted value of 0.0366 1/h [27]. The estimated ${\sigma }_{1} {\text{and}} {\sigma }_{2}$ values are 0.9 and 0.86, respectively. Our model predicts the PK response in both plasma and tight tissues relatively well, but overpredicts the PK in leaky tissues. Moreover, ${\sigma }_{1} {\text{and}} {\sigma }_{2}$ are relatively more sensitive parameter as observed from the sensitivity analysis (Fig. A1). Perturbing ${\sigma }_{1}$ has a reverse effect on the AUC exposure in tight tissues but a direct effect on plasma and leaky tissue exposure. These estimated values of ${\sigma }_{1} {\text{and}} {\sigma }_{2}$ were obtained based on sum of prediction error in plasma, leaky tissues, and tight tissues. As opposed to previous mPBPK models that estimated these reflection coefficients based on plasma concentration. To demonstrate the effect of FcRn binding on PK, we fitted the model against non-specific IgG PK in FcRn knockout mice (Fig. 2) [16]. The estimated ${k}_{up},$ which characterizes the pinocytosis rate into the tissue endosomes, is a rate-limited step for elimination of FcRn non-binding non-specific IgG in plasma and tissues. Therefore, ${k}_{up}$ was recalibrated to 0.15 1/h against FcRn non-binding IgG data [16]. In Fig. A8 in the Supplementary File, we fitted ${k}_{up}$ parameter to the observed plasma concentration of two different mAbs administered in FcRn KO mice [44]. Here, we found ${k}_{up}$ to be 0.26 1/h. Our fitted ${k}_{up}$ parameter for FcRn non-binding mAb varied between 0.15 and 0.26 1/h. The estimated value of ${k}_{up}$ lies between previously reported range, 0.0366 to 1.22 1/h [17]. Also, Davies and Ross measured pinocytosis rate in aortic endothelial cells as 50 nL of fluid pinocytosed per 10^6 cells [45]. Other researchers have used this in vitro estimation to compute endothelial pinocytosis rate in whole body with an estimated number of 6.2 × 10^11 endothelial cells, pinocytosis rate equals 0.031 L/h [46] and pinocytosis rate in different tissues ranging from 0.0003 to 0.065 L/h [43]. The optimized parameters enable the model to fit plasma and tissue PK data for both FcRn non-binding and FcRn binding IgG. In Fig. 3, the model prediction for non-specific FcRn binding IgG was validated using a separate data reported by Baxter et al. for IV dose administered at 3.8 µg [38]. The optimized parameter values are provided in the Supplementary File, Table 1.

### PK of size-variants of a non-specific antibody

**Fig. 4:**
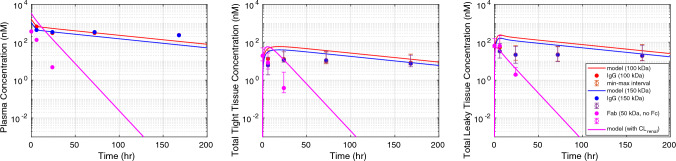
Pharmacokinetic (PK) response of size-variants of a non-specific IgG validated in wild-type mice. The model prediction for non-specific FcRn binding 150 kDa IgG (blue curve) and 100 kDa IgG (red curve) are provided. The concentration in plasma (left), tight (middle), and leaky (right) tissue compartments after intravenous administration of 5 mg/kg dose is shown. The model prediction for non-specific FcRn non-binding 50 kDa IgG (pink curve) concentration in plasma, tight, and leaky tissue compartments after intravenous administration of 5 mg/kg dose is shown. The data intervals are min–max interval interval obtained from the individual tissue concentrations [5] (Color figure online)

The mPBPK model captures the effect of antibody size on plasma and tissue PK. The mPBPK model predicted the PK in plasma and tissues for a full-length IgG (150 kDa) and a one-armed IgG (100 kDa) (Fig. 4) [5]. For larger sized antibodies (> 100 kDa), size-based renal clearance is insignificant. The larger size of these antibodies restricts their movement through size-selective glomerular filtration membrane in the kidneys and other small pores. Our mPBPK predicts slight difference in plasma and tissue PK of 100 kDa IgG and 150 kDa IgG, which is also observed in the reported PK data. Our model demonstrates the plasma and tissue PK of smaller-sized IgG fragments (50 kDa) without an intact Fc region [5]. These smaller sized antibody fragments do not bind to FcRn, and also undergo elimination through the kidneys due to their small size. The inclusion of a renal clearance term in the leaky tissue vasculature (Eq. 4) improved the predicted of leaky tissue concentration and informed the observed PK data for 50 kDa IgG fragment very well. The predicted plasma and tight tissue concentration for 50 kDa IgG fragment did not inform the observed data very well. We believe that incorporating physiology-based and tissue-specific mechanisms of clearance of smaller IgG fragments may be necessary to improve these predictions. Additionally, we also validated the PK of FcRn non-binding antibody fragments with a size ranging between 50 and 100 kDa (Fig. A5) and found that difference between predicted and observed exposure was within 25%. Our mPBPK model successfully predicted the size-based difference in PK in plasma and tissues with some limitations for smaller-size IgG fragments.

### PK of charge-variants of a non-specific antibody

**Fig. 5:**
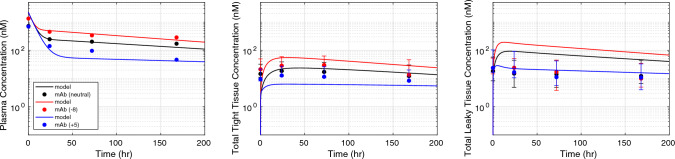
Pharmacokinetic (PK) response of charge-variants of a non-specific IgG in wild-type mice. The model was fitted against three charge-variants of an IgG (150 kDa) for 10 mg/kg IV dose in mice. The predicted concentration in plasma (left), tight (middle), and leaky (right) tissue compartment for a net positive (+ 5) charge-variant (blue curve), neutral charge variant (black curve), and a net negative (− 8) charge variant (red curve) is shown. The data intervals are min–max interval obtained from the individual tissue concentrations [22] (Color figure online)

The effect of charge on antibody PK was evaluated using three charge-variants of IgG (150 kDa), neutral, positive, and negative variants (Fig. 5). The neutral IgG has no net surface charge. The mPBPK model was fitted to neutral IgG PK data, and ${R}_{m,total}$ and ${K}_{D,NSB}$ were estimated to be 71.86 nM and 8.35 nM, respectively. For positive charge variant, model was fitted to data for IgG with a + 5 net surface charge, and ${S}_{pino,2}$, ${K}_{D,NSB}$, and ${K}_{p}$ were estimated to be 2.99, 1.21 nM, and 0.8, respectively. For negative charge variant, model was fitted to IgG with a -8 net surface charge, and ${K}_{D,NSB}$, and ${K}_{p}$ were estimated to be 16.22 nM and 0.62, respectively. A fitted empirical equation between estimated values of ${K}_{D,NSB}$ and ${K}_{p}$ and net surface charge is provided in Fig. A3 and Fig. A4 in the Supplementary File. These quantitative equations are included in the mPBPK model to compute values of ${K}_{D,NSB}$ and ${K}_{p}$ for a given charge. A quadratic function is suitable to denote the relationship between ${K}_{p}$ and charge due to the following reason. Previously reported scaling factors have suggested that volume exclusion is significant for positive and negative charged antibody, which suggests a ${K}_{p}$ value lower than 1. Experimental evidence suggests that ${K}_{p}$ for positive IgG1 is 0.8 and negative IgG4 is 0.4 [17, 34], however these ${K}_{p}$ values were not related to a net antibody charge. Therefore, a re-estimation of these values for charge-dependent data was necessary. The relationship between ${K}_{D,NSB}$ and charge is limited by the data availability for antibody with three charge-variants. A similar trend between ${K}_{D,NSB}$ and charge was reported but no regression fit or equation was provided [22]. However, discrepancies and knowledge gap exist in the quantitative understanding of this relationship [18]. Further, including more descriptive data through experimental validation to augment our comprehension of this correlation between charge and non-specific binding is useful, and currently remains a limitation of the model. The mPBPK model provides a good prediction of the time-dependent concentration of the three charge-variants of IgG in plasma and tight tissues. However, the predicted concentration in leaky tissues is over-predicted for negative and neutral charge-variants of IgG. We found less than onefold error between predicted and observed AUC exposure of the three IgG charge-variants in both plasma and tight tissues. We found a threefold and sixfold error between predicted and observed AUC exposure of the negative and neutral IgG in leaky tissues, respectively. The mPBPK quantitative equations were validated for three separate datasets for IgG with net charge of − 4, − 10, and + 10 as shown in Fig. A6 in the Supplementary File.

### Target-mediated disposition of a specific antibody

**Fig. 6:**
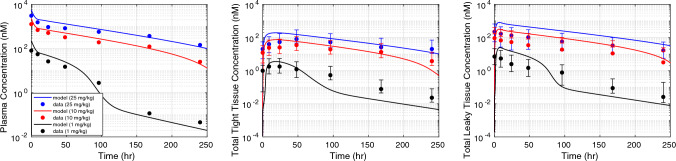
Target-mediated drug disposition of an anti-CEA IgG (150 kDa) after intravenous (IV) administration of 1 mg/kg, 10 mg/kg, and 25 mg/kg dose in mice. The model fitting results of anti-CEA IgG administered at 1 mg/kg dose (black curve) are compared against observed concentration in plasma (left), tight tissue (middle), leaky tissue (right) (solid black points) [37]. The model prediction of anti-CEA IgG concentration in plasma and tissues was validated for IV dose administered at 10 mg/kg (red curve) and 25 mg/kg (blue curve) against observed data [37]. The data intervals are min–max interval obtained from the individual tissue concentrations (Color figure online)

The effects of CEA antigen on antibody PK are shown through our mPBPK model. Figure 6 captures the fitted model prediction (black curve) of an anti-CEA IgG in plasma and tissues for an intravenous (IV) dose of 1 mg/kg in mice [37]. The estimated internalization rate ($${k}_{int} $$) of membrane-bound mAb-antigen complex is 0.015 1/h. The estimated internalization rate is close to the degradation rate of membrane-bound antigen (0.019 1/h). The half-life of membrane-bound antigen is estimated to be 36 h. The baseline concentration is based on literature values and set for soluble antigens ($${T}_{s0 }$$) to 2 ng/ml [36] and membrane-bound antigens to 80 nM [35]. We validated the target-mediated disposition of anti-CEA IgG in mice against observed plasma and tissue concentrations [37]. We found that fitting these two parameters helped in capturing the target-mediated disposition of drug in plasma. The predicted concentration deviates from the observed data in tight and leaky tissues. The predicted PK response can be improved further by calibrating other sensitive model parameters governing drug-target dynamics. The validation was performed for an anti-CEA IgG administered at 10 mg/kg IV dose and 25 mg/kg IV dose [37]. The model predicts TMDD in both plasma and tissues at these higher doses very well. Besides, we acknowledge that incorporating other use cases with different antigens and antigen properties should help improve model robustness towards TMDD prediction.

### Predicting the effect of target affinity and charge on disposition of antibody in humans

**Fig. 7:**
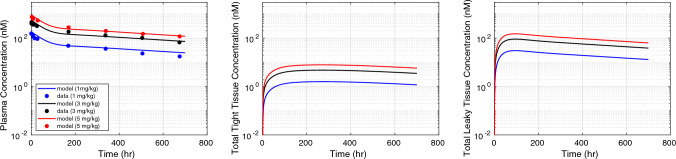
Target-mediated drug disposition of an anti-TNF-α IgG (148 kDa) after intravenous (IV) administration of 1 mg/kg, 3 mg/kg, and 5 mg/kg in human subjects (70 kg). Plasma concentration validated against observed data using human mPBPK model (left). Total tight (middle) and leaky (right) tissue concentrations are predicted over 700 h for respective doses

**Fig. 8:**
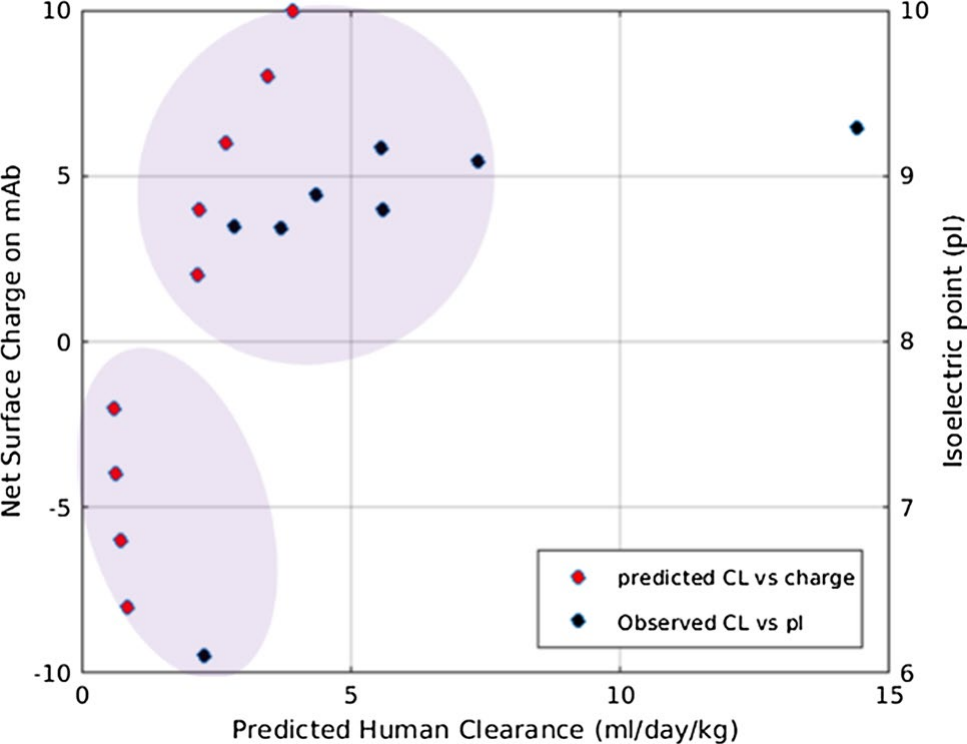
Predicted overall plasma clearance from human mPBPK model. The linear clearance (CL= kel_(terminal slope)_*V_p_/BW) (red points) was calculated from plasma PK simulated for a series of charge variants (− 8 to + 10) of an antibody (~ 148 kDa) using human mPBPK model. The correlation between observed human clearance and Isoelectric point (pI) of an antibody as reported by Zheng et al. [48] is digitized and shown here (black points). kel_(terminal slope)_ is the rate of elimination (1/h) calculated from the terminal slope of the PK profile. V_p_  is the volume of plasma. BW is the body weight (70 kg) (Color figure online)

To demonstrate the capability of our model in characterizing the plasma and tissue disposition of mAb in humans, we simulated the model with a priori known physiology-based parameters and kinetic rate constants for humans. The human physiology-based parameters were obtained from Shah et al. [27] and Delanaye et al. [47] and kinetic parameters such as $k_{up} , CL_{rec} , CL_{cat} , FcRn_{Total} , k_{on} , k_{off} , k_{1on} , k_{1off} , \,{\text{and}}\,CL_{p,T}$ were obtained from Yuan et al. [24]. Kinetic parameters whose values are not known are kept same as the mice mPBPK model. We simulated the mPBPK model against observed plasma PK of adalimumab in human (70 kg), an anti-TNF-α antibody. The baseline concentration of soluble TNF-α receptor was set to 0.276 pM and degradation rate of TNF-α receptor was set to 8.316 L/h as reported previously [24]. As shown in Fig. 7, we observe a good agreement between model predicted plasma concentration of adalimumab administered at three different IV bolus doses (1 mg/kg, 3 mg/kg, and 5 mg/kg) [24]. The predicted total concentrations in tight and leaky tissues are also provided in Fig. 7. We also checked the efficiency of the human mPBPK model towards predicting charge-dependent PK. Due to lack of clinical studies with mAb charge-variants, we qualitatively compared the predicted PK of two mAbs with pI = 6 with net negative charge and pI = 9 with net positive charge, as shown in Fig. A7 in Supplementary File. We calculated the linear plasma clearance (L/h) of both mAb1 (pI = 6) and mAb2 (pI = 9) from the terminal slope of plasma PK. We found a 0.75-fold change in linear clearance between mAb1 with lower pI and mAb2 with a higher pI. Zheng et al. reported observed human clearance of 0.0062 L/h for an mAb with pI = 6.1 and 0.0131 L/h for an mAb with pI = 9.4, which is a 0.53-fold change in clearance values [48]. Zheng et al. also provided a correlation between observed plasma clearance in humans for different mAbs and their pI values. To demonstrate a similar correlation between charge and clearance, we calculated linear clearance of different charge-variants of mAb from their predicted human plasma PK. We assumed different net surface charge on these mAbs ranging from − 8 to + 10, and simulated their plasma PK response. We compared the predicted correlation between charge and clearance against reported correlation between pI and clearance (Fig. 8). We found our predicted correlation was qualitatively similar to the correlation between observed human clearance (ml/day/kg) and pI reported by Zheng et al. [48]. There is a clear shift in predicted and observed plasma clearance for increasing charge or pI values (Fig. 8).

## Discussion

Monoclonal antibody pharmacokinetics is an extensively studied area with increasingly more focus on their ADME properties. To study the relationship between antibody properties and ADME, PBPK models are often the models of choice since they provide quantitative descriptions of the drug disposition process in a biological system that can be scaled between species based on physiological differences [27]. In this study, we built a mPBPK model that incorporates such quantitative descriptions between antibody properties and essential ADME processes involved in the PK of an antibody. Published whole-body and minimal PBPK models have been previously developed to describe the ADME of antibodies using the physicochemical properties of antibodies such as size and FcRn binding affinity [5, 9, 10, 16, 27]. To our knowledge, a combined effect of various physicochemical properties of antibodies such as MW, size, charge, binding affinities to FcRn, and specific targets have not been included in a single mPBPK model previously. In our mPBPK model, we lumped different tissue compartments with similar kinetics into one compartment. Such lumping of compartments benefits in dimensionality and complexity reduction while allowing successful representation of a whole-body PBPK model [26]. However, other reduced PBPK models have been modeled with more sophistication than others, where tissue compartments are lumped based on different capillary structure in different sub-spaces in the tissues such as vascular space and interstitial space [41]. Based on the research problem, we believe that refining the model structure, including additional sub-compartments, and predicting specific tissue concentration may be necessary. However, these model improvements for precise prediction of tissue distribution of mAbs are often subject to data availability.

The mPBPK model developed in this work incorporates the interaction between a monoclonal antibody and FcRn receptor. The FcRn receptor is an essential mechanism that is characterized in our mPBPK model and plays an important role in the selection and design of antibodies for desired PK properties. Previous PBPK models have accounted for this mAb-FcRn interaction with varying levels of complexities. FcRn receptors are expressed in a variety of tissues such as the renal proximal tubules, endothelial cells of the muscle vasculature, monocytes, intestinal macrophages, dendritic cells [27]. In our mPBPK model, we include FcRn receptor in the endosomal space within endothelial cells of tight and leaky tissues, and in the endosomal space in the systemic vascular endothelial cells nested to the plasma compartment. Including another site of FcRn interaction adds another dimension of complexity to the model but allows us to investigate the biological impact of the receptor at different sites. Our study captured the role of mAb-FcRn binding in non-specific mAbs (Fig. 2). FcRn-binding mAbs showed lower clearance compared to mAbs that did not bind to FcRn (Fig. 2). Besides FcRn binding, there are other factors that affect mAb elimination such as pinocytosis, proteolysis, target-mediated effects etc. The rate of pinocytosis was the rate-limiting step in our mPBPK model, which meant recalibrating the rate to capture mAb PK in FcRn knockout mice. It has been shown FcRn-mediated elimination route is not saturable for mAbs, therefore FcRn binding is not expected to impact mAb PK alone when non-specific clearance is dominant [1, 27]. Our mPBPK successfully demonstrates the behavior of FcRn binding and non-binding on antibody pharmacokinetics.

Among the physicochemical properties, the effect of protein size has been studied extensively in the past. It is well known that the transcapillary movement of proteins or large molecules through different tissue vasculature is size-dependent. The two-pore hypothesis allows the quantitative description of this transcapillary transport of protein molecules. Several published PBPK models have successfully incorporated the two-pore model related parameters [9–11]. The size-dependent effects of large molecule drugs were added by using the two-pore hypothesis in our mPBPK model. There are two distinct phases of microvascular wall permeability that depend on the protein size [11]. The permeability falls steeply below a molecular size of 3.5 nm, which represents the limits of the small pores, whereas the permeability remains almost constant at a molecular size greater than 5 nm [11]. Therefore, a system of small and large pores based on the two-pore theory, act as a route for convective transfer of different sized antibodies in our model. Our model showed that the monoclonal antibodies with a molecular size range between 4.5 and 5 nm (100–150 kDa) had much slower clearance when compared to antibody fragments (~ 50 kDa) (Fig. 4). The large molecule drugs (150 kDa) are almost completely restricted by small pores and have relatively low permeability through large pores. Additionally, the size-selective glomerular membranes in the kidney contribute towards the relatively fast clearance of smaller-sized antibody fragments. We provide an equation to calculate renal clearance of smaller sized antibody fragments as a function of physiology based GFR and glomerular sieving coefficient (Eq. 4). The glomerular sieving coefficient changes with antibody size (Stoke’s radius) as shown in Fig. A2. One of the limitations of our model is that it captures the size-dependent effect on PK in a limited range of MW between 50 and 150 kDa, whereas other whole body PBPK models have demonstrated PK prediction for proteins with MW between 13 and 150 kDa [10].

The variability in antibody PK among different antibody modalities with comparable values of MW, size, and FcRn binding affinity is attributed to other physicochemical factors, including molecular charge. Recent studies are focused on studying the effect of net charge, charge distribution, and isoelectric point on antibody plasma and tissue PK [22, 23, 33]. Our mPBPK model adapts a similar framework to incorporate the effect of net surface charge on PK. In this framework, we estimate the change in pinocytosis, change in interstitial volume of distribution, and non-specific binding affinity to putative receptors that collectively arise from charge-based interactions. The charge-dependent effect on pinocytosis rate in different tissues [22, 32, 43] and non-specific interactions [4, 22, 33] of a drug with the cell components has been proven previously but has generally been difficult to relate to drug disposition or predictive outcome in a mechanistic way due to the lack of large datasets and diversity in types of measures defining charge or non-specific interaction. The charge-dependent effect on volume of distribution of mAbs within the interstitial space in the tissues was demonstrated in skin and muscle tissues [25, 34]. An approximation for fraction of interstitial volume available for a slightly positive mAb and a slightly negative mAb was found to be 0.8 and 0.4, respectively [34]. These previous analyses were useful in quantitatively relating charge with essential PK processes in the model. It is often challenging to obtain the pinocytosis rate of clearance and volume of interstitial distribution in individual tissues for different mAb charge-variants. Similarly, it is difficult to experimentally measure the equilibrium dissociation rate constant for various non-specific charge-based interaction between mAb and the cell components. Therefore, these parameters were fitted to various charge-variants of an antibody and an empirical relationship was proposed (Fig. A2, A3). These proposed quantitative relationships may be helpful in predicting PK of different drug modalities. Using these relationships for a limited dataset, our mPBPK model predicted slower clearance of negative and neutral charged mAb, whereas faster plasma clearance of positively charged mAb (Fig. 5), which agrees with the dataset source [22]. In contrast, studies reported a monotonic or uncorrelated relationship between clearance and extreme charge variants of antibody, that is extreme negative charge on an antibody increased its clearance in plasma [18]. On the other hand, studies reported that extreme positive charge patches on antibody led to aggregation and failed analysis [18]. Possible explanations were suggested for the uncorrelation between clearance and extremely negative charge that include changes in FcRn binding, other physicochemical properties like hydrophobicity, and/or certain tissues may recognize and take up extremely negative mAbs from systemic circulation [18, 49]. Thus, limited by the available observed PK data for different mAb charge-variants, our mPBPK model incorporates the quantitative relation to PK for antibodies within a limited range of net charge between − 10 and + 10. This is another limitation of our model and can be overcome by additional good quality data for different antibody charge-variants. Moreover, the mPBPK model over-predicted the concentration in leaky tissues for negative and neutral charge-variants of mAb. We believe that physiological parameters such as and may be responsible for the overprediction in leaky tissues, which are fixed based on either approximation or estimation. The reflection coefficient (σ_2_) represent the level of convective resistance of the pores in the tissue vasculature to the antibody. Reflection coefficients are often approximated or estimated using observed data to achieve predefined levels of antibody available in tissue/plasma [11]. Moreover, can vary between subject, species, and antibodies [11]. Tissue lymph flows (L_1_, L_2_) are often well approximated from plasma flow into tissues. However, re-calibrating these values using observed tissue PK data is not uncommon [9, 38, 50] and may improve our model predictions further. We also used the human physiology-based model to demonstrate a qualitative but significant correlation between linear plasma clearance and antibody charge (Fig. 8). It has been reported that most antibodies are slightly positively charged with pI values between 7 and 9.10 [6]. Our predicted correlation shows clear distinction in clearance values for mAb with positive charge and mAb with negative charge for charge variations in the range − 8 to + 8 (Fig. A7). This observation is similar to reported observed clearance values in humans, where higher overall clearance is observed for mAbs with pI between 8 and 10 [48].

One of the objectives of our mPBPK model is to account for drug’s engagement with a specific antigen, which is driven by the drug’s physicochemical properties. Our model predicts a given drug’s target engagement response when information about the drug and the antigen is provided (Fig. 6). We included essential processes that drive antibody and target interaction. Our mPBPK demonstrated a good prediction of target-mediated PK through a case study of anti-CEA IgG in mice (Fig. 6). The quality and accuracy of target-mediated effect on PK can be used to evaluate and compare various drugs and gain insight on their physicochemical properties. Moreover, our mPBPK model characterized the plasma PK of adalimumab in humans very well (Fig. 7). The mPBPK model predicts target engagement based on predicted concentration of mAb-antigen complex and total antigen. However, in this study we do not consider the pharmacodynamic effect when developing the mPBPK model which is a notable limitation of our model. At present, the model only accounts for change in target baseline values and real-time changes in target concentration. The model has no bias between types of receptors and accounts for various forms of receptors (soluble or membrane-bound). This allows the mPBPK model to be customized when different target expression and target forms are present specific to an antibody in question. The model does not explicitly account for change in cell type or cell numbers, however more sophisticated additions to the existing model specific to disease/problem of interest may address this limitation.

The mPBPK model can serve as a platform model-based approach to identify drug properties that influence PK and target engagement. There are several easy to use software platforms such as PK-Sim, GastroPlus, and Sim-cyp available for PBPK modeling of mAbs, which offer similar levels of model complexity. Each of these software predicted accurate serum concentrations but predicted ranges of tissue concentration due to differences in parameterization and assumptions [51]. The mPBPK model does have an added advantage over other readily available commercial software, as it includes the effect of net antibody charge on ADME characteristics besides other properties like MW/size, FcRn/Antigen binding. Another limitation with user-friendly software was found to be the lack of clear description of assumptions, input parameters, model structure, and calculations in the available documentation [51]. On the other hand, the mPBPK model may offer limited tissue-specific prediction and site-specific effect in tissues but these predictions can be improved through necessary model additions and use of descriptive data at effect-site to improve the prediction accuracy of the tissue of interest for the research problem of interest.

## Conclusions

The mPBPK model developed in this work incorporates a multivariate quantitative relationship between physicochemical properties of large molecule drugs and their PK. Our mPBPK model takes in physiology-based species parameters and drug-specific properties such as molecular weight, molecular size (Stoke’s radius), molecular charge, binding affinity to FcRn, and specific target affinity. Through derived and fitted empirical relations between drug properties and PK parameters of the model, we predicted the change in PK response in both plasma and tissues of different drug modalities. Our mPBPK model shows promise as a tool to predict plasma PK and exposure in not only pre-clinical species such as mice but also clinical subjects. In the future, this mPBPK model is planned to be used as a tool to evaluate and compare PK of different drugs with varying physicochemical properties. Additionally, we aim to incorporate descriptive data to augment our comprehension of the correlation between ADME properties, such as charge and non-specific binding, and their consequent impact on drug disposition outcomes in pre-clinical species and humans. The proposed minimal PBPK model can contribute to a better understanding of the biology-specific PK and ADME processes of therapeutic proteins and serve as a platform model-based approach to identify drug properties mostly influence PK and target engagement.

## Supplementary Information

Below is the link to the electronic supplementary material.

Supplementary file1 (DOCX 306 KB)
