# Supplementary material for: A minimal physiologically based pharmacokinetic model to study the combined effect of antibody size, charge, and binding affinity to FcRn/antigen on antibody pharmacokinetics
Source: J Pharmacokinet Pharmacodyn. 2024 Feb 24;51(5):477–92. doi: 10.1007/s10928-023-09899-z (PMC11576895; doi:10.1007/s10928-023-09899-z)
Supplement: Supplementary file 1 — Supplementary file1 (DOCX 306 KB) [file 10928_2023_9899_MOESM1_ESM.docx]

A Minimal Physiologically based Pharmacokinetic Model to Study the Combined Effect of Antibody Size, Charge, and Binding Affinity to FcRn/Antigen on Antibody pharmacokinetics.

Krutika Patidar^1^, Nikhil Pillai^2^, Saroj Dhakal^2^, Lindsay B. Avery^3^, Panteleimon D. Mavroudis^2, *^

Affiliations:

^1^University at Buffalo, School of Chemical and Biochemical Engineering, Buffalo, NY, USA

^2^Global DMPK Modeling & Simulation, Sanofi, Cambridge, MA, USA

^3^Global DMPK Innovation, Sanofi, Cambridge, MA, USA

^*^Corresponding author:

Panteleimon D. Mavroudis

350 Water St, Cambridge, MA-02141, USA

[panteleimon.mavroudis@sanofi.com](mailto:panteleimon.mavroudis@sanofi.com)

# Supplementary Information

## Model Equations:

*Plasma compartment*

$\frac{dA_{p}}{\mathrm{dt}}=\mathrm{CL}_{\mathrm{rec}} \mathrm{FcRnA}_{e}/V_{p}-{S_{\mathrm{pino}}*k}_{up,p} A_{p}+(L A_{l}- (\text{1-}\sigma_{1}\text{)}L_{1}\text{ }A_{p} - \text{(1-}\sigma_{2}\text{)}L_{2}\text{ }A_{p})/V_{p}-k_{\mathrm{on}}A_{p}T_{p}+k_{\mathrm{off}}\mathrm{ATC}_{p}$ (A1)

$\frac{dT_{p}}{\mathrm{dt}}=k_{\mathrm{syn}}-k_{\mathrm{on}}A_{p}T_{p}+k_{\mathrm{off}}\mathrm{ATC}_{p} -{(k}_{p,T} -{S_{\mathrm{pino}}*k}_{up,p} )T_{p} - {S_{\mathrm{pino}}*k}_{up,p}T_{p}$ (A2)

$\frac{d\mathrm{ATC}_{p}}{\mathrm{dt}}= {- k}_{\mathrm{on}}A_{p}T_{p}+k_{\mathrm{off}}\mathrm{ATC}_{p} +(L \mathrm{ATC}_{l}-\left( 1-\sigma_{1} \right)L_{1}\mathrm{ATC}_{p}- \text{(1}-\sigma_{2}\text{)}L_{2}\text{ }\mathrm{ATC}_{p})/V_{p}+\mathrm{CL}_{\mathrm{rec}} \mathrm{FcRnATC}_{e}/V_{p}-{S_{\mathrm{pino}}*k}_{up,p} \mathrm{ATC}_{p}$ (A3)

*Plasma* *nested* *endosomes*

$\frac{dA_{e}}{\mathrm{dt}}=S_{\mathrm{pino}}*k_{\mathrm{up}}*A_{p}+k_{1off} \mathrm{FcRnA}_{e}-k_{1on}A_{e}\mathrm{FcRn}_{e}\text{ }-k_{\deg} A_{e}\text{ }-\mathrm{ke}_{\mathrm{on}}A_{e}T_{e}+\mathrm{ke}_{\mathrm{off}}\mathrm{ATC}_{e}$ (A4)

$\frac{dT_{e}}{\mathrm{dt}}=-\mathrm{ke}_{\mathrm{on}}A_{e}T_{e}+\mathrm{ke}_{\mathrm{off}}\mathrm{ATC}_{e}-\mathrm{ke}_{\mathrm{on}}\mathrm{FcRnA}_{e}T_{e}+\mathrm{ke}_{\mathrm{off}}\mathrm{FcRn}\mathrm{ATC}_{e} -\mathrm{CL}_{\mathrm{cat}}T_{e}/V_{e}+S_{\mathrm{pino}}*k_{\mathrm{up}}T_{v}$ (A5)

$\frac{d\mathrm{ATC}_{e}}{\mathrm{dt}}={S_{\mathrm{pino}}*k}_{\mathrm{up}}*\mathrm{AT}C_{e}+k_{1off} \mathrm{FcRnATC}_{e}-k_{1on}\mathrm{ATC}_{e}\mathrm{FcRn}_{e}\text{ }-\mathrm{CL}_{\mathrm{cat}} \mathrm{ATC}_{e}/V_{e}+\mathrm{ke}_{\mathrm{on}}A_{e}T_{e}-\mathrm{ke}_{\mathrm{off}}\mathrm{ATC}_{e}$ (A6)

$\frac{d\mathrm{FcRn}_{e}}{\mathrm{dt}}=-k_{1on}A_{e}\mathrm{FcRn}_{e}+k_{1off} \mathrm{FcRnA}_{e}-k_{1on}\mathrm{ATC}_{e}\mathrm{FcRn}_{e}+k_{1off} \mathrm{FcRnATC}_{e}+\mathrm{CL}_{\mathrm{rec}}*(\mathrm{FcRnA}_{e}+{\mathrm{FcR}\mathrm{nATC}}_{e})/V_{e}$ (A7)

$\frac{d\mathrm{FcRnA}_{e}}{\mathrm{dt}}=k_{1on}A_{e}\mathrm{FcRn}_{e}-k_{1off} \mathrm{FcRnA}_{e}-\mathrm{ke}_{\mathrm{on}}\mathrm{FcRnA}_{e}T_{e}+ \mathrm{ke}_{\mathrm{off}}\mathrm{FcRn}\mathrm{ATC}_{e} -\mathrm{CL}_{\mathrm{rec}}\mathrm{FcRnA}_{e}/V_{e}$ (A8)

$\frac{d\mathrm{FcRnATC}_{e}}{\mathrm{dt}}=k_{1on}\mathrm{ATC}_{e}\mathrm{FcRn}_{e}-k_{1off} \mathrm{FcRnATC}_{e}+\mathrm{ke}_{\mathrm{on}}\mathrm{FcRnA}_{e}T_{e}-\mathrm{ke}_{\mathrm{off}}\mathrm{FcRn}\mathrm{ATC}_{e} -\mathrm{CL}_{\mathrm{rec}}\mathrm{FcRnATC}_{e}/V_{e}$ (A9)

*Tight Tissue (vascular)*

$\frac{dA_{v1}}{\mathrm{dx}} = (\text{1-}\sigma_{1}\text{)}L_{1}\text{ }A_{p}/{(K_{p}\mathrm{Vv}}_{1})- \mathrm{CL}_{TP1}A_{v1}/{(K_{p}\mathrm{Vv}}_{1})+FR{*\mathrm{CL}}_{\mathrm{rec}}\mathrm{FcRnA}_{e1}/{(K_{p}\mathrm{Vv}}_{1})- {S_{pino,1}*k}_{\mathrm{up}}A_{v1}{- k}_{\mathrm{on}}A_{v1}T_{v1}+k_{\mathrm{off}}\mathrm{ATC}_{v1} - kon_{\mathrm{nsb}}*A_{v1}*\left( Rm_{\mathrm{tot}} \right)+ koff_{\mathrm{nsb}}*A\mathrm{Rm}_{v1}- kon*A_{v1}*\mathrm{Tm}_{v1} + koff*A\mathrm{Tm}_{v1}$ (A10)

$\frac{d\mathrm{ATC}_{v1}}{\mathrm{dx}} = (\text{1-}\sigma_{1}\text{)}L_{1}\text{ }\mathrm{ATC}_{p}/{(K_{p}\mathrm{Vv}}_{1})+FR{*\mathrm{CL}}_{\mathrm{rec}}\mathrm{FcRnATC}_{e1}/{(K_{p}\mathrm{Vv}}_{1})- {S_{pino,1}*k}_{\mathrm{up}}\mathrm{ATC}_{v1}+k_{\mathrm{on}}A_{v1}T_{v1}-k_{\mathrm{off}}\mathrm{ATC}_{v1}$ (A11)

$\frac{dT_{v1}}{\mathrm{dt}}=k_{\mathrm{syn}}-k_{\mathrm{on}}A_{v1}T_{v1}+k_{\mathrm{off}}\mathrm{ATC}_{v1} -{(k}_{p,T} -S_{pino,1}*k_{\mathrm{up}} )T_{v1} -S_{pino,1}*k_{\mathrm{up}}T_{v1}$ (A12)

$\frac{d\mathrm{Tm}_{v1}}{\mathrm{dt}}=k_{syn.m}-k_{\mathrm{on}}A_{v1}\mathrm{Tm}_{v1}+k_{\mathrm{off}}\mathrm{ATC}_{v1} -k_{p,T} Tm_{v1}$ (A13)

$\frac{\mathrm{dA}\mathrm{Rm}_{v1}}{\mathrm{dt}}=\mathrm{kon}_{\mathrm{nsb}}A_{v1}RM_{\mathrm{tot}}-\mathrm{koff}_{\mathrm{nsb}}\mathrm{ARm}_{v1}$ (A14)

$\frac{\mathrm{dA}\mathrm{Tm}_{v1}}{\mathrm{dt}}=k_{\mathrm{on}}A_{v1}Tm_{v1}-k_{\mathrm{off}}\mathrm{ATm}_{v1}-k_{\mathrm{int}}\mathrm{ATm}_{v1}$ (A15)

*Tight Tissue (endosomes)*

$\frac{dA_{e1}}{\mathrm{dt}}=S_{\mathrm{pino}}*k_{\mathrm{up}}*A_{v1}+k_{1off} \mathrm{FcRnA}_{e1}-k_{1on}A_{e1}\mathrm{FcRn}_{e1}\text{ }-k_{\deg} A_{e1}-\mathrm{ke}_{\mathrm{on}}A_{e1}T_{e1}+\mathrm{ke}_{\mathrm{off}}\mathrm{ATC}_{e1}$

(A16)

$\frac{dT_{e1}}{\mathrm{dt}}=-\mathrm{ke}_{\mathrm{on}}A_{e1}T_{v1}+\mathrm{ke}_{\mathrm{off}}\mathrm{ATC}_{e1}-\mathrm{ke}_{\mathrm{on}}\mathrm{FcRnA}_{e1}T_{e1}+\mathrm{ke}_{\mathrm{off}}\mathrm{FcRn}\mathrm{ATC}_{e1} -\mathrm{CL}_{\mathrm{cat}}T_{e1}/V_{e1}+S_{pino,1}*k_{\mathrm{up}}T_{v1}$

(A17)

$\frac{d\mathrm{ATC}_{e1}}{\mathrm{dt}}={S_{pino,1}*k}_{\mathrm{up}}*\mathrm{AT}C_{v1}+k_{1off} \mathrm{FcRnATC}_{e1}-k_{1on}\mathrm{ATC}_{e1}\mathrm{FcRn}_{e1}\text{ }-\mathrm{CL}_{\mathrm{cat}} \mathrm{ATC}_{e1}/V_{e1}+\mathrm{ke}_{\mathrm{on}}A_{e1}T_{e1}-\mathrm{ke}_{\mathrm{off}}\mathrm{ATC}_{e1}$ (A18)

$\frac{d\mathrm{FcRn}_{e1}}{\mathrm{dt}}=-k_{1on}A_{e1}\mathrm{FcRn}_{e1}+k_{1off} \mathrm{FcRnA}_{e1}-k_{1on}\mathrm{ATC}_{e1}\mathrm{FcRn}_{e1}+k_{1off} \mathrm{FcRnATC}_{e1}+\mathrm{CL}_{\mathrm{rec}}*(\mathrm{FcRnA}_{e1}+\mathrm{FcRnATC}_{e1})/V_{e1}$ (A19)

$\frac{d\mathrm{FcRnA}_{e1}}{\mathrm{dt}}=k_{1on}A_{e1}\mathrm{FcRn}_{e1}-k_{1off} \mathrm{FcRnA}_{e1}-\mathrm{ke}_{\mathrm{on}}\mathrm{FcRnA}_{e1}T_{e1}+ \mathrm{ke}_{\mathrm{off}}\mathrm{FcRn}\mathrm{ATC}_{e1} -\mathrm{CL}_{\mathrm{rec}}\mathrm{FcRnA}_{e1}/V_{e1}$

(A20)

$\frac{d\mathrm{FcRnATC}_{e1}}{\mathrm{dt}}=k_{1on}\mathrm{ATC}_{e1}\mathrm{FcRn}_{e1}-k_{1off} \mathrm{FcRnATC}_{e1}+\mathrm{ke}_{\mathrm{on}}\mathrm{FcRnA}_{e1}T_{e1}-\mathrm{ke}_{\mathrm{off}}\mathrm{FcRn}\mathrm{ATC}_{e1} -\mathrm{CL}_{\mathrm{rec}}\mathrm{FcRnATC}_{e1}/V_{e1}$ (A21)

*Tight tissue (interstitium)*

$\frac{dA_{\mathrm{is}1}}{\mathrm{dt}}=-\frac{\left( 1- \sigma_{L} \right)L_{1}A_{\mathrm{is}1}}{\left( K_{p}V_{\mathrm{is}1} \right)}-S_{pino,1}*k_{\mathrm{up}}A_{\mathrm{is}1} +\frac{\left( 1 -\mathrm{FR} \right)\mathrm{CL}_{\mathrm{rec}}\mathrm{FcRnA}_{e1}}{K_{p}V_{\mathrm{is}1}}+\mathrm{CL}_{\mathrm{TP}1}A_{\mathrm{is}1}/(K_{p}V_{\mathrm{is}1})$ (A22)

$\frac{d\mathrm{ATC}_{\mathrm{is}1}}{\mathrm{dt}}=- \left( 1- \sigma_{L} \right)L_{1}\mathrm{ATC}_{\mathrm{is}1}/(K_{p}V_{\mathrm{is}1}) - {S_{pino,1}*k}_{\mathrm{up}}\mathrm{ATC}_{\mathrm{is}1} + (1 -\mathrm{FR})\mathrm{CL}_{\mathrm{rec}}\mathrm{FcRnATC}_{e1}/(K_{p}V_{\mathrm{is}1})$(A23)

*Leaky Tissue (vascular)*

$\frac{dA_{v2}}{\mathrm{dx}} = (\text{1-}\sigma_{2}\text{)}L_{2}\text{ }A_{p}/{(K_{p}\mathrm{Vv}}_{2})- {(\mathrm{CL}_{\mathrm{renal}}+ CL}_{TP2}{)A}_{v2}/{(K_{p}\mathrm{Vv}}_{2})+FR{*\mathrm{CL}}_{\mathrm{rec}}\mathrm{FcRnA}_{e2}/{(K_{p}\mathrm{Vv}}_{2})- {S_{pino,2}*k}_{\mathrm{up}}A_{v2}{- k}_{\mathrm{on}}A_{v2}T_{v2}+k_{\mathrm{off}}\mathrm{ATC}_{v2} - kon_{\mathrm{nsb}}*A_{v2}*\left( Rm_{\mathrm{tot}} \right)+ koff_{\mathrm{nsb}}*A\mathrm{Rm}_{v2}- kon*A_{v2}*\mathrm{Tm}_{v2} + koff*A\mathrm{Tm}_{v2}$ (A24)

$\frac{d\mathrm{ATC}_{v2}}{\mathrm{dx}} = (\text{1-}\sigma_{2}\text{)}L_{2}\text{ }\mathrm{ATC}_{p}/{(K_{p}\mathrm{Vv}}_{2})+FR{*\mathrm{CL}}_{\mathrm{rec}}\mathrm{FcRnATC}_{e2}/{(K_{p}\mathrm{Vv}}_{2})- {S_{\mathrm{pin}o,2}*k}_{\mathrm{up}}\mathrm{ATC}_{v2}+k_{\mathrm{on}}A_{v2}T_{v2}-k_{\mathrm{off}}\mathrm{ATC}_{v2}$ (A25)

$\frac{dT_{v2}}{\mathrm{dt}}=k_{\mathrm{syn}}-k_{\mathrm{on}}A_{v2}T_{v2}+k_{\mathrm{off}}\mathrm{ATC}_{v2} -{(k}_{p,T} -S_{pino,2}*k_{\mathrm{up}} )T_{v2} -S_{pino,2}*k_{\mathrm{up}}T_{v2}$ (A26)

$\frac{d\mathrm{Tm}_{v2}}{\mathrm{dt}}=k_{syn.m}-k_{\mathrm{on}}A_{v2}\mathrm{Tm}_{v2}+k_{\mathrm{off}}\mathrm{ATC}_{v2} -k_{p,T} Tm_{v2}$ (A27)

$\frac{\mathrm{dA}\mathrm{Rm}_{v2}}{\mathrm{dt}}=\mathrm{kon}_{\mathrm{nsb}}A_{v2}RM_{\mathrm{tot}}-\mathrm{koff}_{\mathrm{nsb}}\mathrm{ARm}_{v2}$ (A28)

$\frac{\mathrm{dA}\mathrm{Tm}_{v2}}{\mathrm{dt}}=k_{\mathrm{on}}A_{v2}Tm_{v2}-k_{\mathrm{off}}\mathrm{ATm}_{v2}-k_{\mathrm{int}}\mathrm{ATm}_{v2}$ (A29)

*Leaky Tissue (endosomes)*

$\frac{dA_{e2}}{\mathrm{dt}}=S_{pino,2}*k_{\mathrm{up}}*A_{v2}+k_{1off} \mathrm{FcRnA}_{e2}-k_{1on}A_{e2}\mathrm{FcRn}_{e2}\text{ }-k_{\deg} A_{e2}\text{ }-\mathrm{ke}_{\mathrm{on}}A_{e2}T_{e2}+\mathrm{ke}_{\mathrm{off}}\mathrm{ATC}_{e2}$

(A30)

$$\frac{dT_{e2}}{\mathrm{dt}}=-\mathrm{ke}_{\mathrm{on}}A_{e2}T_{v2}+\mathrm{ke}_{\mathrm{off}}\mathrm{ATC}_{e2}-\mathrm{ke}_{\mathrm{on}}\mathrm{FcRnA}_{e2}T_{e2}+\mathrm{ke}_{\mathrm{off}}\mathrm{FcRn}\mathrm{ATC}_{e2} -\frac{\mathrm{CL}_{\mathrm{cat}}T_{e2}}{V_{e2}}+S_{pino,2}*k_{\mathrm{up}}T_{v2}$$

(A31)

$\frac{d\mathrm{ATC}_{e2}}{\mathrm{dt}}={S_{pino,2}*k}_{\mathrm{up}}*\mathrm{AT}C_{v2}+k_{1off} \mathrm{FcRnATC}_{e2}-k_{1on}\mathrm{ATC}_{e2}\mathrm{FcRn}_{e2}\text{ }-\mathrm{CL}_{\mathrm{cat}} \mathrm{ATC}_{e2}/V_{e2}+\mathrm{ke}_{\mathrm{on}}A_{e2}T_{e2}-\mathrm{ke}_{\mathrm{off}}\mathrm{ATC}_{e2}$ (A32)

$\frac{d\mathrm{FcRn}_{e2}}{\mathrm{dt}}=-k_{1on}A_{e2}\mathrm{FcRn}_{e2}+k_{1off} \mathrm{FcRnA}_{e2}-k_{1on}\mathrm{ATC}_{e2}\mathrm{FcRn}_{e2}+k_{1off} \mathrm{FcRnATC}_{e2}+\mathrm{CL}_{\mathrm{rec}}*(\mathrm{FcRnA}_{e2}+\mathrm{FcRnATC}_{e2})/V_{e2}$ (A33)

$\frac{d\mathrm{FcRnA}_{e2}}{\mathrm{dt}}=k_{1on}A_{e2}\mathrm{FcRn}_{e2}-k_{1off} \mathrm{FcRnA}_{e2}-\mathrm{ke}_{\mathrm{on}}\mathrm{FcRnA}_{e2}T_{e2} \mathrm{ke}_{\mathrm{off}}\mathrm{FcRn}\mathrm{ATC}_{e2} -\mathrm{CL}_{\mathrm{rec}}\mathrm{FcRnA}_{e2}/V_{e2}$

(A34)

$\frac{d\mathrm{FcRnATC}_{e2}}{\mathrm{dt}}=k_{1on}\mathrm{ATC}_{e2}\mathrm{FcRn}_{e2}-k_{1off} \mathrm{FcRnATC}_{e2}+\mathrm{ke}_{\mathrm{on}}\mathrm{FcRnA}_{e2}T_{e2}-\mathrm{ke}_{\mathrm{off}}\mathrm{FcRn}\mathrm{ATC}_{e2} -\mathrm{CL}_{\mathrm{rec}}\mathrm{FcRnATC}_{e2}/V_{e2}$ (A35)

*Leaky tissue (interstitium)*

$\frac{dA_{\mathrm{is}2}}{\mathrm{dt}}=- \left( 1- \sigma_{L} \right)L_{2}A_{\mathrm{is}2}/(K_{p}V_{\mathrm{is}2}) -S_{pino,2}*k_{\mathrm{up}}A_{\mathrm{is}2} + (1 -\mathrm{FR})\mathrm{CL}_{\mathrm{rec}}\mathrm{FcRnA}_{e2}/(K_{p}V_{\mathrm{is}2})+ \mathrm{CL}_{\mathrm{TP}2}A_{\mathrm{is}2}/(K_{p}V_{\mathrm{is}2})$ (A36)

$\frac{d\mathrm{ATC}_{\mathrm{is}2}}{\mathrm{dt}}=- \left( 1- \sigma_{L} \right)L_{2}\mathrm{ATC}_{\mathrm{is}2}/(K_{p}V_{\mathrm{is}2}) - {S_{pino,2}*k}_{\mathrm{up}}\mathrm{ATC}_{\mathrm{is}2} + (1 -\mathrm{FR})\mathrm{CL}_{\mathrm{rec}}\mathrm{FcRnATC}_{e2}/(K_{p}V_{\mathrm{is}2})$ (A37)

*Lymph compartment*

$\frac{dA_{l}}{\mathrm{dt}}=\left( 1-\sigma_{L} \right)*L1*\frac{A_{is1}}{V_{L}}+\left( 1-\sigma_{L} \right)*L2*\frac{A_{is2}}{V_{L}} -L*\frac{A_{l}}{V_{L}}$ (A38)

$\frac{d\mathrm{ATC}_{l}}{\mathrm{dt}}=\left( 1-\sigma_{L} \right)*L1*\frac{\mathrm{ATC}_{is1}}{V_{L}}+\left( 1-\sigma_{L} \right)*L2*\frac{\mathrm{ATC}_{is2}}{V_{L}} -L*\frac{\mathrm{ATC}_{l}}{V_{L}}$ (A39)

| State variable | Description |
| --- | --- |
| $A_{p}$ | Drug (A) concentration in plasma |
| $T_{p}$ | Target (T) concentration in plasma |
| $\mathrm{ATC}_{p}$ | Drug-target complex (ATC) in plasma |
| $A_{e}$ | Drug (A) in nested endosomes |
| $T_{e}$ | Target (T) in nested endosomes |
| $\mathrm{ATC}_{e}$ | Drug-target complex (ATC) in nested endosomes |
| $\mathrm{FcRn}_{e}$ | FcRn concentration in nested endosomes |
| $\mathrm{FcRnA}_{e}$ | FcRn-drug complex concentration in nested endosomes |
| $\mathrm{FcRnATC}_{e}$ | FcRn-drug-target complex concentration in nested endosomes |
| $A_{v1}, A_{v2}$ | Drug (A) concentration in tight (1) vasculature or leaky (2) vasculature |
| $T_{v1}, T_{v2}$ | Target (T) concentration in tight (1) vasculature or leaky (2) vasculature |
| $\mathrm{ATC}_{v1}, \mathrm{ATC}_{v2}$ | Drug-target complex (ATC) concentration in tight (1) vasculature or leaky (2) vasculature |
| $\mathrm{Tm}_{v1}, \mathrm{Tm}_{v2}$ | Membrane-bound Target (Tm) concentration in tight (1) vasculature or leaky (2) vasculature |
| $\mathrm{ATm}_{v1}, \mathrm{ATm}_{v2}$ | Membrane-bound Drug-target complex (ATm) concentration in tight (1) vasculature or leaky (2) vasculature |
| $\mathrm{ARm}_{v1}, \mathrm{ARm}_{v1}$ | Drug-receptor protein complex (ARm) concentration in tight (1) vasculature or leaky (2) vasculature |
| $A_{e1}, A_{e2}$ | Drug (A) concentration in tight (1) or leaky (2) endosomes |
| $T_{e1}, T_{e2}$ | Target (T) concentration in tight (1) or leaky (2) endosomes |
| $\mathrm{ATC}_{e1}, \mathrm{ATC}_{e2}$ | Drug-target complex (ATC) concentration in tight (1) or leaky (2) endosomes |
| $\mathrm{FcRn}_{e1}, FcRn_{e2}$ | FcRn concentration in tight (1) or leaky (2) endosomes |
| $\mathrm{FcRnA}_{e1}, \mathrm{FcRnA}_{\mathrm{ei}}$ | FcRn-drug complex concentration in tight (1) or leaky (2) endosomes |
| $\mathrm{FcRnATC}_{e1}, FcRnATC_{e2}$ | FcRn-drug-target complex concentration in tight (1) or leaky (2) endosomes |
| $A_{is1}, A_{is2}$ | Drug (A) concentration in tight (1) or leaky (2) interstitial space |
| $\mathrm{ATC}_{\mathrm{is}1}, {ATC}_{is2}$ | Drug-target complex (ATC) concentration in tight (1) or leaky (2) interstitial space |
| $A_{l}$ | Drug (A) concentration in lymph compartment |
| $\mathrm{ATC}_{l}$ | Drug-target (ATC) complex in lymph compartment |

*Calculation for two-pore mediated clearance*

$J_{l,1}=J_{iso,1}+ (\alpha_{l})*L1$ (A40)

$J_{s,1}=J_{iso,1}+ ({1 - \alpha}_{l})*L1$ (A41)

$J_{l,2}=J_{iso,2}+ (\alpha_{l})*L2$ (A42)

$J_{s,2}=J_{iso,2}+ ({1 - \alpha}_{l})*L2$ (A43)

$a_{e}=0.0483*\mathrm{MW}^{0.386}$ (A44)

$\sigma_{l}= 0.000035*\mathrm{MW}^{0.717}$ (A45)

$\sigma_{s}= 1 - 0.8489*e^{-0.00004*MW}$ (A46)

$J_{l,i}, J_{s,i},$are lymph flow rate through large and small pores, respectively. $a_{e}$ is the Stoke’s radius of a protein. $\sigma_{l}, \sigma_{s}$ are vascular reflection coefficient of large and small pores, respectively. Molecular weight (MW) is in g/mol or Dalton units. Here, i = 1 represents tight tissues and i =2 represents leaky tissues.

Two-pore-clearance rate ($CL_{\mathrm{TP}}$) via large and small pores

$CL_{TP_{L},i}=PS_{L,i}\left( 1- \frac{C_{is, i}}{C_{v,i}} \right) \frac{Pe_{L}}{e^{Pe_{L}}-1}+J_{L,i}\left( 1- \sigma_{L} \right)$ (A47)

$CL_{TP_{S},i}=PS_{S,i}\left( 1- \frac{C_{is,i}}{C_{v,i}} \right) \frac{Pe_{S}}{e^{Pe_{S}}-1}+J_{S,i}\left( 1- \sigma_{S} \right)$ (A48)

$PS_{L,i}, PS_{S,i}, Pe_{L}, Pe_{S}, \sigma_{L}, \sigma_{S}$ were calculated using derived equations by Li et al. (supplementary file [1]). Here, i = 1 represents tight tissues and i =2 represents leaky tissues.

**Table 1: Physiology-based and kinetic parameters for mice. Blank cells denote that parameter values were dependent on antibody data.**

| Parameters | Definition | Value (Mice) | Reference | Value  (Human) | Reference |
| --- | --- | --- | --- | --- | --- |
| $\mathbf{BW}$ | Body weight | 0.028 kg | Shah 2012 | 70 kg | [2] |
| $\mathbf{V}_{\mathbf{p}}$ | Volume of plasma | 0.85 ml | Shah 2012 | 2.6 L | [2] |
| $\mathbf{V}_{\mathbf{L}}$ | Volume of lymph compartment | 1.717 ml | Shah 2012 | 5.2 L | [2] |
| $\mathbf{L}_{\mathbf{1}}$ | Lymph flow rate in tight tissue | 2.7820E-04 L/h | Cao 2013 | 0.039 L/h | [2] |
| $\mathbf{L}_{\mathbf{2}}$ | Lymph flow rate in leaky tissue | 4.6792E-04 L/h | Cao 2013 | 0.081 L/h | [2] |
| $\mathbf{L}$ | Lymph flow rate | 0.000738 L/h | Yuan 2018 | 0.12 L/h | [2] |
| $\mathbf{V}_{\mathbf{e}}$ | Endosomal volume in systemic vascular endothelial cells. | 0.005/100*BW | Yuan 2018 | 0.005/100*BW | [2] |
| $\mathbf{V}_{\mathbf{e1}}$ | Volume of endosome in tight tissues | 0.09404 ml | Shah 2012 | 0.285 L | [3] |
| $\mathbf{V}_{\mathbf{e2}}$ | Volume of endosome in leaky tissues | 0.03685 ml | Shah 2012 | 0.056 L | [3] |
| $\mathbf{V}_{\mathbf{v1}}$ | Volume of vascular space in tight | 0.4695 ml | Shah 2012 | 0.968 L | [3] |
| $\mathbf{V}_{\mathbf{v2}}$ | Volume of vascular space in leaky | 0.3477 ml | Shah 2012 | 0.745 L | [3] |
| $\mathbf{V}_{\mathbf{1}}$ | Volume of interstitial fluid volume in tight | 3.554 ml | Shah 2012 | 10.14 L | [3] |
| $\mathbf{V}_{\mathbf{2}}$ | Volume of interstitial fluid volume in leaky | 1.3539 ml | Shah 2012 | 5.46 L | [3] |
| $\boldsymbol{\sigma}_{\mathbf{1}}$ | Reflection coefficient | 0.9 | *estimated* | 0.94 | [2] |
| $\boldsymbol{\sigma}_{\mathbf{2}}$ | Reflection coefficient | 0.86 | *estimated* | 0.69 | [2] |
| $\mathbf{FR}$ | Fraction recycled in vascular space | 0.715 | Shah 2012 | 0.715 | [3] |
| $\mathbf{FcR}\mathbf{n}_{\mathbf{total}}$ | Total FcRn concentration | 40 $\mu M$ | Shah 2012 | 49.8 $\mu M$ | [2] |
| $\mathbf{GFR}$ | Glomerular filtration rate | 0.0167 L/h | Shah 2012 | -- |  |
| $\mathbf{k}_{\mathbf{up}\mathbf{,}\mathbf{p}}$ | Uptake rate | 0.05 1/h | *estimated* | 0.0617 L/h | [2] |
| $\mathbf{k}_{\mathbf{up}}$ | Uptake rate | 0.027 1/h | *estimated* | 0.0617 L./h | [2] |
| $\mathbf{CL}_{\mathbf{rec}}$ | Recycling clearance rate in plasma endosomes | 7.27E-06 | Yuan 2018 | 0.0182 L./h | [2] |
| $\mathbf{k}_{\mathbf{deg}}$ | Degradation rate of free drug through catabolic pathways | 42.9 1/h | Shah 2012 | 0.24 L/h | [2] |
| $\mathbf{CL}_{\mathbf{cat}}$ | catabolic clearance rate of drug-target complex assumed same as target | 1.295 ml/day | Yuan 2018 | 0.24 L/h | [2] |
| $\mathbf{k}\mathbf{1}_{\mathbf{on}}$ | Association rate constant for drug-FcRn binding | 0.12 1/nM.h | Yuan 2018 | 0.867 1/nM.h | [2] |
| $\mathbf{k}\boldsymbol{1}_{\mathbf{off}}$ | Dissociation rate constant for drug-FcRn binding | 1.8 1/h | Yuan 2018 | 583 1/h | [2] |
| $\mathbf{k}_{\mathbf{on}}$ | Association rate constant for drug-target binding at physiological pH (7.4) | 5.9 1/nM.h | Ferl 2005 | 2.59 1/nM.h | [2] |
| $\mathbf{k}_{\mathbf{off}}$ | Dissociation rate constant for drug-target binding at physiological pH (7.4) | 0.0508 1/h | Ferl 2005 | 0.468 1/h | [2] |
| $\mathbf{k}\mathbf{e}_{\mathbf{on}}$ | Association rate constant for drug-target binding at endosomal pH (6) | 5.904 1/h | Ferl 2005 | 2.59 1/nM.h | [2] |
| $\mathbf{k}\mathbf{e}_{\mathbf{off}}$ | Dissociation rate constant for drug-target binding at endosomal pH (6) | 0.0508 1/h | Ferl 2005 | 0.468 1/h | [2] |
| $\mathbf{k}_{\mathbf{p}\mathbf{,}\mathbf{T}}$ | Rate of degradation of soluble target | 0.3465 1/h | *estimated* | 8.31 L/h | [2] |
| $\mathbf{k}_{\mathbf{p}\mathbf{,}\mathbf{Tm}}$ | Rate of degradation of membrane-bound target | 0.0192 1/h | *estimated* | -- | -- |
| $\mathbf{IC}\mathbf{C}_{\mathbf{p,T}}$ | Baseline concentration of soluble target | 0.016 nM | Kiyama 1990 | 2.76E-4 nM | [2] |
| $\mathbf{IC}\mathbf{C}_{\mathbf{p,Tm}}$ | Baseline concentration of membrane-bound target | 80 nM | Ferl 2005 | -- | -- |
| $\mathbf{k}_{\mathbf{syn}}$ | Rate of synthesis of soluble target | 0.0055 nM/h | *calculated* | 0.0023 nM/h | [2] |
| $\mathbf{k}_{\mathbf{syn}\mathbf{,}\mathbf{m}}$ | Rate of synthesis of membrane-bound target | 1.53 nM/h | *calculated* | -- | -- |
| $\mathbf{k}_{\mathbf{int}}$ | Internalization rate of membrane-bound drug-target complex | 0.015 1/h | *estimated* | -- | -- |
| $\mathbf{R}_{\mathbf{m}\mathbf{,}\mathbf{total}}$ | Total negatively charged receptor concentration | 71.86 nM | *estimated* | 71.86 nM | *estimated* |

Note: L = L1 + L2. $\mathbf{CL}_{\mathbf{rec}}$ is calculated using transit time (8 min) and volume of endosomes.
$\mathbf{k}_{\mathbf{syn}}$ = $\mathbf{IC}\mathbf{C}_{\mathbf{p,T}}$ *$\mathbf{k}_{\mathbf{p,T}}$ for soluble receptors. $\mathbf{k}_{\mathbf{syn,m}}$ = $\mathbf{IC}\mathbf{C}_{\mathbf{p,Tm}}$ *$\mathbf{k}_{\mathbf{p,Tm}}$ for membrane-bound receptors.

**Table 2: Two-pore transport model related parameters used in eq. A40 - A48 for both mouse and human physiology.**

| Parameters | Definition | Value | Reference |
| --- | --- | --- | --- |
| $\mathbf{r}_{\mathbf{l}}$ | Large pore radius | 22.85 nm | [1] |
| $\mathbf{r}_{\mathbf{s}}$ | Small pore radius | 4.44 nm | [1] |
| $\mathbf{D}$ | Diffusion coefficient | 1.92e-5 cm2/min | [1] |
| $\boldsymbol{\eta}$ | Viscosity | 1.12e-9 N*min/cm2 | [1] |
| $\boldsymbol{\alpha}_{\mathbf{l}}$ | Fraction of hydraulic conductivity | 0.042 | [1] |
| $\mathbf{J}_{\mathbf{iso}\mathbf{,}\mathbf{tight}}$ | recirculation flow rate (L/h) | 0.38*L1 | [1] |
| $\mathbf{J}_{\mathbf{iso}\mathbf{,}\mathbf{leaky}}$ | recirculation flow rate (L/h) | 0.38*L2 | [1] |

Table 3: Model Assumptions and Limitations

| Index | Model Assumptions | Reference |
| --- | --- | --- |
| 1 | FcRn concentration in tissue and plasma endosomes remains same but varies with species. | [3] |
| 2 | Administered mAb are assumed to bind to FcRn at only slightly acidic, pH=6, in the endosomes. | [2] |
| 3 | Soluble targets are synthesized both in plasma and in vascular space in the tissues. | [2] |
| 4 | Membrane-bound targets are synthesized in the tissue vascular space. | [2] |
| 5 | Membrane-bound mAb-antigen complex internalize at the same rate as the rate of antigen degradation. | [4] |
| 6 | The drug target complex and unbound target are catabolized at the same rate ($CL_{cat}$). | [2] |
| 7 | The mAb-antigen complex internalized in the tissues, and the internalized molecules are assumed to be metabolize further but are not explicitly modeled. |  |
| 8 | Tissue vasculature is porous, and the radius of pores is divided into small pores (40 nm) and large pores (220 nm) | [1] |
| 9 | Total concentration of membrane protein receptor ($R_{m,total}$) is fixed across tissues. | [5] |
| 10 | The non-specific binding ($k_{on,NSB}$) between mAb and membrane proteins is equal to the mAb-FcRn association rate constant ($k_{1on}$). | [6] |
| 11 | Antibody-antigen association ($k_{on}$) and dissociation rate ($k_{off}$) constant are same for soluble and membrane-bound antigens. | [7] |
|  |  |  |
| Index | **Model Limitations** |  |
| 1 | Size-dependent effects are limited within a MW range between 50-150 kDa. |  |
| 2 | Charge-dependent effects are limited within a range of net charge between -10 to + 10 |  |
| 3 | No cellular space considered to describe downstream processes. |  |
| 4 | Model offers limited tissue-specific and site-specific prediction in tissues. |  |
| 5 | No Pharmacodynamic effects are considered in the current model |  |

## Supplementary Figures


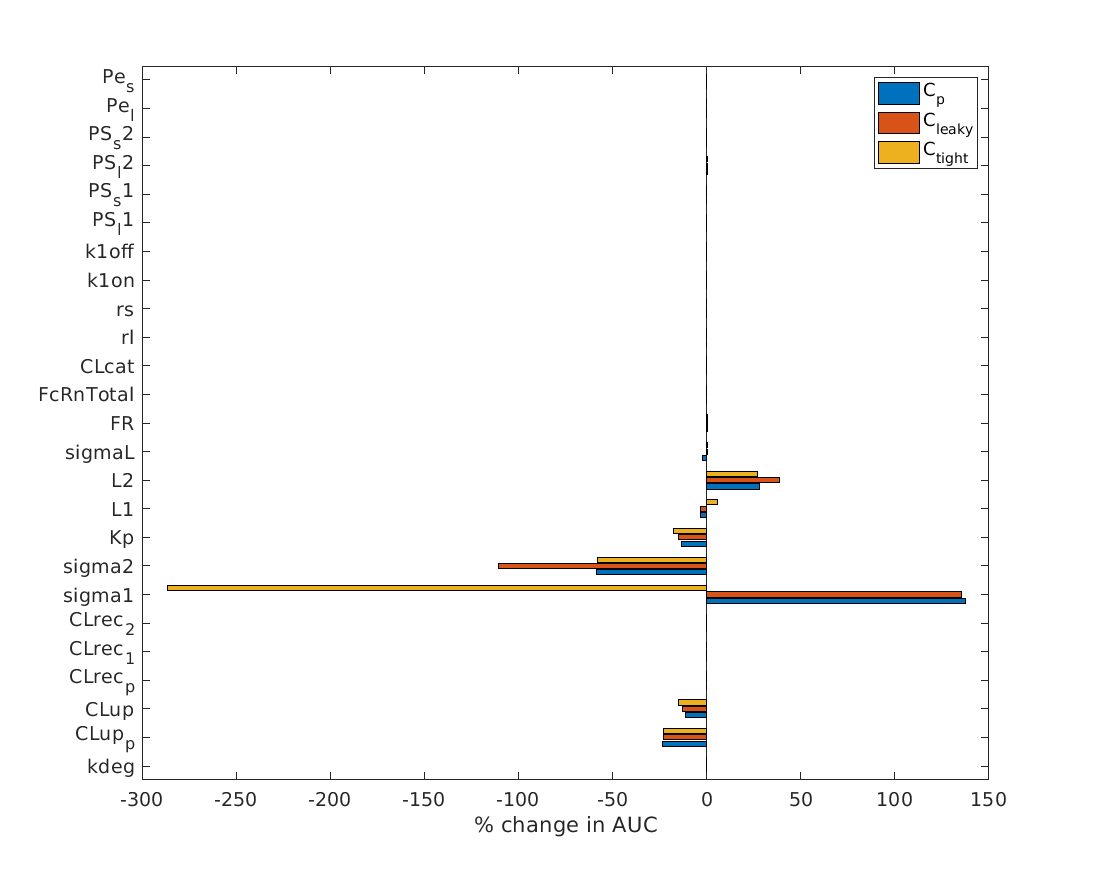


Fig. A1: Local sensitivity analysis of model parameters. The horizontal bars depict a percent change in AUC (horizontal axis) of plasma, tight tissue, and leaky tissue concentration for 20% increase in respective parameter values.


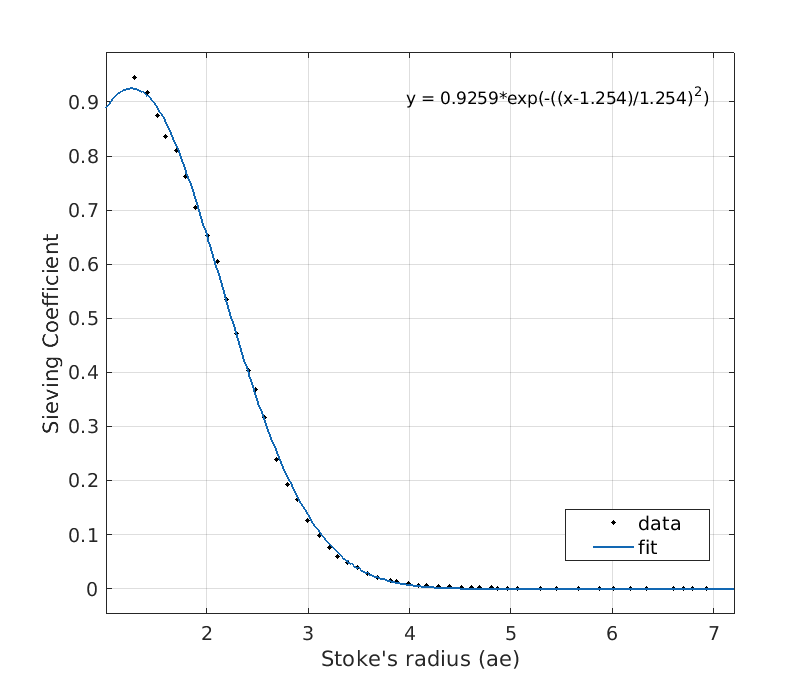


Fig. A2: Empirical relation between sieving coefficient (vertical axis) and Stoke’s radius of an antibody (horizontal axis) obtained from curve fitting in MATLAB. The fitted equation is shown, where y is the sieving coefficient and x is the Stoke’s radius or antibody size. Sum of squared error for the fit is 0.002678 and R-squared is 0.999.


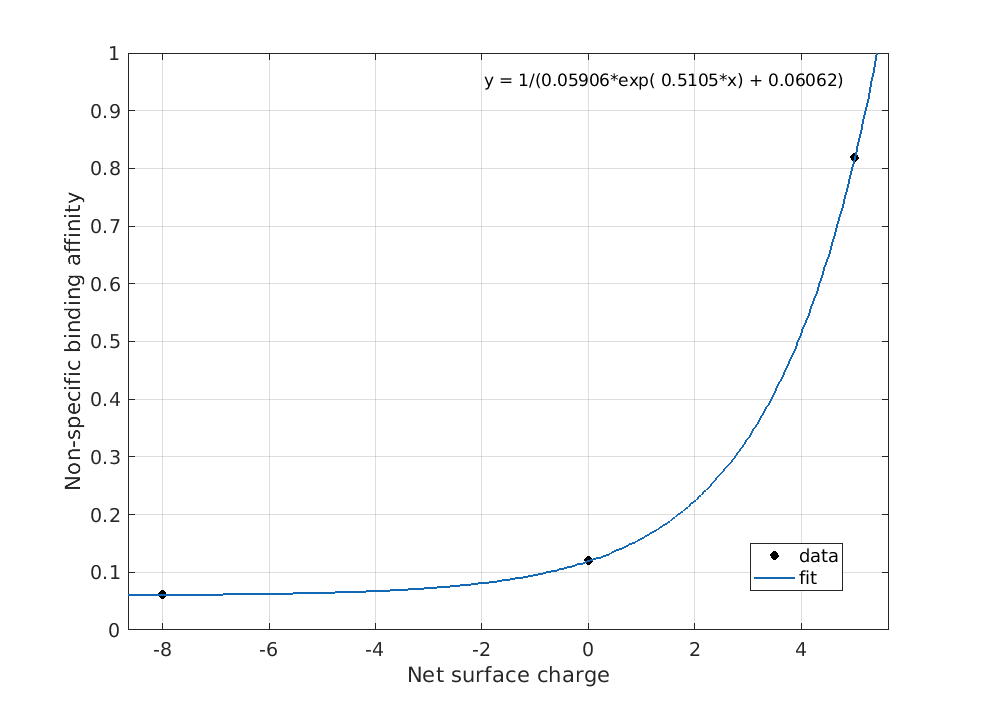


Fig. A3: Empirical relation between non-specific binding affinity (1/$\mathbf{K}_{\mathbf{D,NSB}}$) (vertical axis) and net surface charge (horizontal axis) on an antibody obtained from curve fitting in MATLAB. The fitted equation is shown, where y is the non-specific binding affinity and x is the net surface charge. The sum of squared error for the is 3.711e-13 and R-squared is 1.


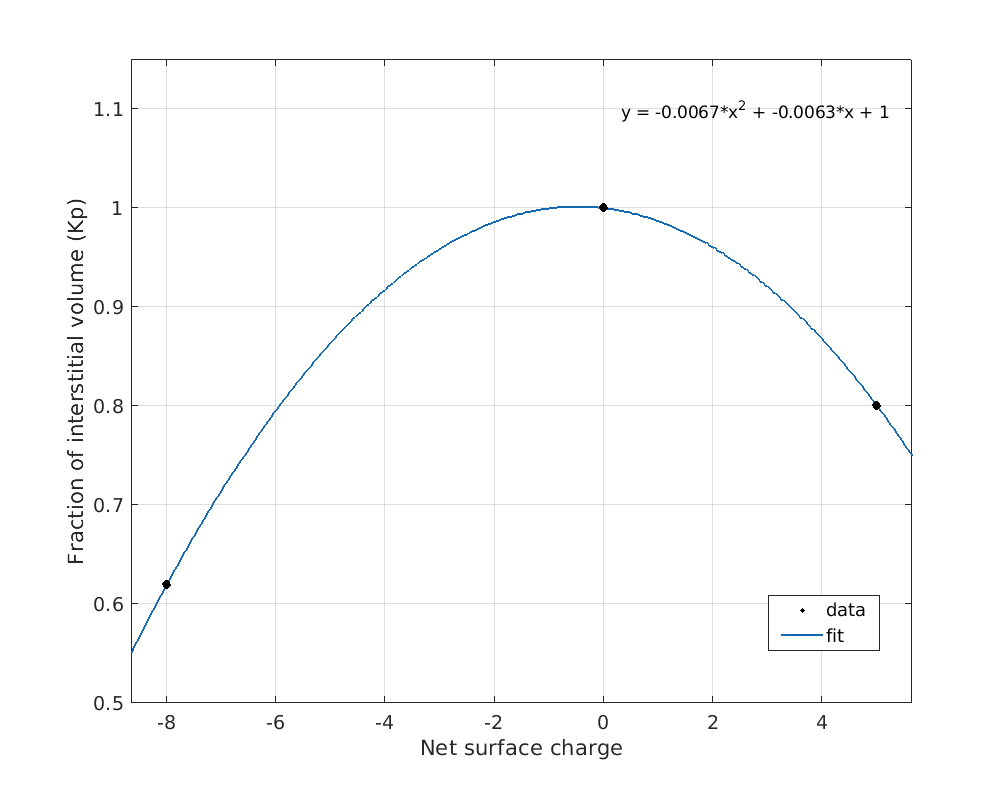


Fig. A4: Empirical relation between modulation factor ($\mathbf{K}_{\mathbf{p}}$) (vertical axis) and net surface charge (horizontal axis). Modulation factor ($\mathbf{K}_{\mathbf{p}}$) denotes the fraction of interstitial volume available for antibody distribution. The fitted equation is shown, where y is the modulation factor $\mathbf{K}_{\mathbf{p}}$and x is the net surface charge. The sum of squared error is 1.233e-32 and R-squared is 1.


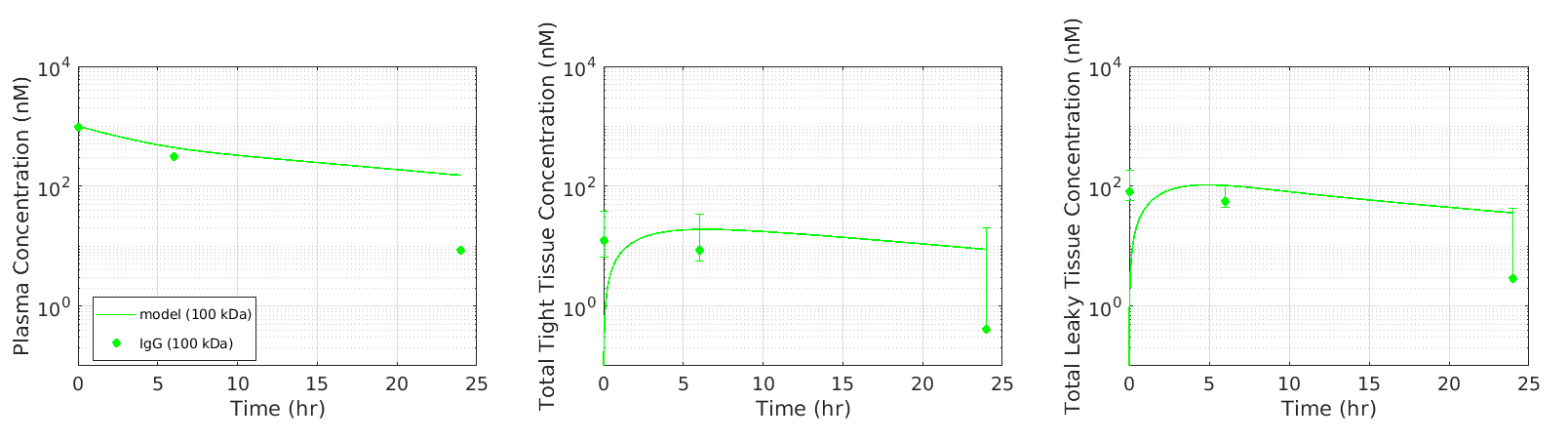


Fig. A5: The effect of size of an antibody on plasma (left), tight tissue (middle), and leaky tissue (right) concentration. The concentration time profile of IgG fragment Fab2 (100 kDa) is predicted. Data for antibody fragment, Fab2, was obtained from Rafidi et al. [8]. The data intervals are min-max interval available from individual tissue concentration data.


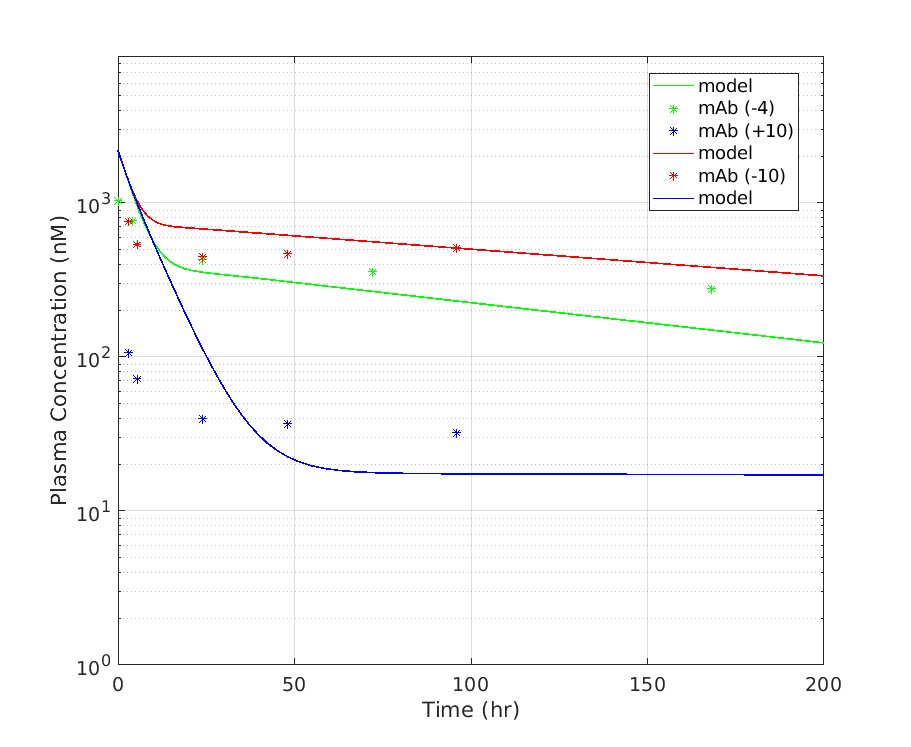


Fig. A6: Pharmacokinetic (PK) response of charge-variants of a non-specific IgG validated in wild-type mice. The model prediction for three charge-variants of an IgG (150 kDa) after intravenous administration of 5 mg/kg [9] and 10 mg/kg [9] in mice is shown. The predicted concentration in plasma (left), tight (middle), and leaky (right) tissue compartment for a net negative (-4) charge-variant (pink curve) is shown against observed data (pink) [9]. The predicted concentration with a net positive (+10, blue) and net negative (-10, red) charge is also compared against observed data [9]. The data intervals are min-max interval obtained from the individual tissue concentrations.


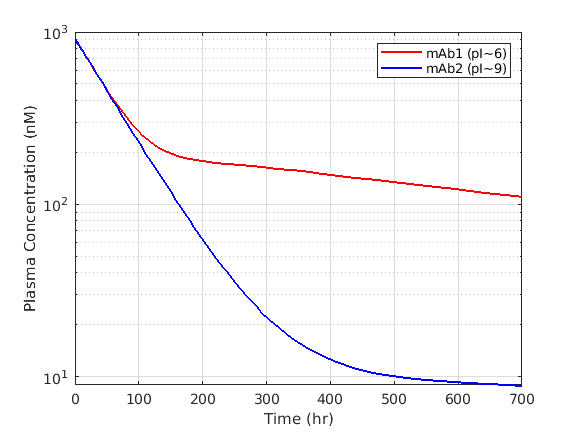


Fig. A7: Pharmacokinetic (PK) response of monoclonal antibody (mAb) with different isoelectric points (pI) validated in using mPBPK model for humans. The plasma concentration of mAb1 (pI ~ 6) with a hypothetical negative charge (-8) was predicted for an IV bolus dose of 5 mg/kg (red). The plasma concentration of mAb2 (pI ~ 9) with a hypothetical positive charge (+8) was predicted for an IV bolus dose of 5 mg/kg (blue). The calculated clearance (L/h) = $\mathbf{ke}\mathbf{l}_{\mathbf{terminal slope}}\mathbf{*V}$ for mAb1 was 0.0025 L/h and for mAb2 was 0.0101 L/h.


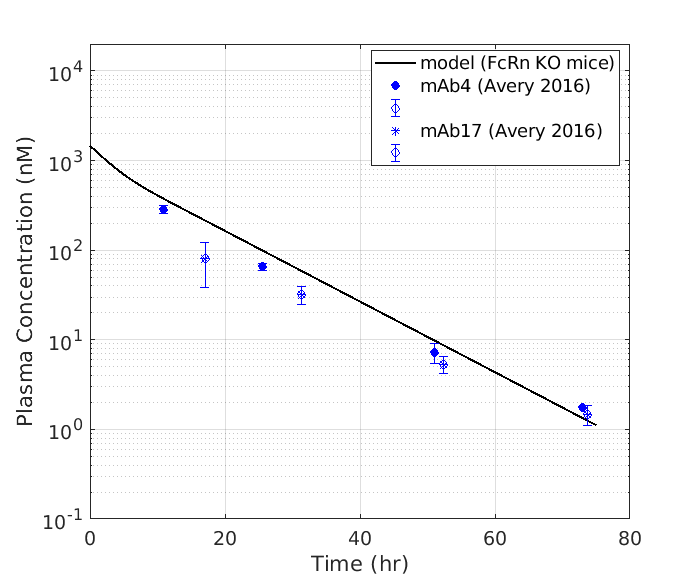


Fig. A8: Mouse mPBPK model fitted to plasma concentration of two mAbs (mAb4 and mAb17). The model is fitted to observed plasma concentration of mAb4 (blue points) and mAb17 (asterisk) in FcRn KO mice [10]. $\boldsymbol{k}_{\boldsymbol{up}}$ was re-calibrated for this dataset and found to be 0.26 1/h.

# References

| [1] | Z. Li and D. K. Shah, "Two-pore physiologically based pharmacokinetic model with de novo derived parameters for predicting plasma PK of different size protein therapeutics.," *Journal of pharmacokinetics and pharmacodynamics,* pp. 305-218, 2019. |
| --- | --- |
| [2] | D. Yuan, F. Rode and Y. Cao, "A Minimal Physiologically Based Pharmacokinetic Model with a Nested Endosome Compartment for Novel Engineered Antibodies.," *The AAPS journal,* 2018. |
| [3] | D. K. Shah and A. M. Betts, "Towards a platform PBPK model to characterize the plasma and tissue disposition of monoclonal antibodies in preclinical species and human.," *Journal of pharmacokinetics and pharmacodynamics,* p. 67–86., 2012. |
| [4] | M. Ovacik and K. Lin, "Tutorial on Monoclonal Antibody Pharmacokinetics and Its Considerations in Early Development," *Clin Transl Sci,* vol. 11, 2018. |
| [5] | S. Liu and D. K. Shah, "Physiologically Based Pharmacokinetic Modeling to Characterize the Effect of Molecular Charge on Whole-Body Disposition of Monoclonal Antibodies.," *AAPS Journal,* 2023. |
| [6] | H. M. Jones, Z. Zhang, P. Jasper, H. Luo, L. B. Avery, L. E. King, H. Neubert, H. A. Barton, A. M. Betts and R. Webster, ", A Physiologically-Based Pharmacokinetic Model for the Prediction of Monoclonal Antibody Pharmacokinetics From In Vitro Data," *CPT Pharmacometrics Syst. Pharmacol.,* 2019. |
| [7] | G. Z. Ferl, A. M. Wu and J. J. DiStefano, "A predictive model of therapeutic monoclonal antibody dynamics and regulation by the neonatal Fc receptor (FcRn).," *Annals of biomedical engineering,* 2005. |
| [8] | H. Rafidi, S. Rajan, K. Urban, Shatz, W. Shatz-Binder, K. Hui, G. Z. Ferl, A. V. Kamath and C. A. Boswell, "Effect of molecular size on interstitial pharmacokinetics and tissue catabolism of antibodies," *mAbs,* 2022. |
| [9] | J. C. Stüber, K. F. Rechberger, S. M. Miladinović, T. Pöschinger, T. Zimmermann, R. Villenave, M. J. Eigenmann, T. E. Kraft, D. K. Shah, H. Kettenberger and W. F. Richter, "Impact of charge patches on tumor disposition and biodistribution of therapeutic antibodies," *AAPS Open,* 2022. |
| [10] | L. B. Avery, M. Wang, M. S. Kavosi, A. Joyce, J. C. Kurz, Y. Y. Fan, M. E. Dowty, M. Zhang, Y. Zhang, A. Cheng, F. Hua, H. M. Jones, H. Neubert, R. J. Polzer and D. M. O'Hara, "Utility of a human FcRn transgenic mouse model in drug discovery for early assessment and prediction of human pharmacokinetics of monoclonal antibodies.," *MAbs,* vol. 8, pp. 1064-78, 2016. |
